# Phytochemical Diversity and Antioxidant Potential of *Dracocephalum* Species: Current Knowledge and Future Perspectives

**DOI:** 10.3390/antiox15060771

**Published:** 2026-06-19

**Authors:** Madalina Georgiana Pantazi, Oana Cioanca, Ionut Iulian Lungu, Catalin Tanase, Silvia Robu, Denisa Batir-Marin, Monica Hancianu

**Affiliations:** 1 Faculty of Pharmacy, Grigore T. Popa University of Medicine and Pharmacy, 16 University Street, 700115 Iasi, Romania; madalina-georgiana.pantazi@d.umfiasi.ro (M.G.P.); ionut-iulian.lungu@umfiasi.ro (I.I.L.); monica.hancianu@umfiasi.ro (M.H.); 2 Faculty of Biology, Alexandru Ioan Cuza University Carol I 20A, 700505 Iasi, Romania; tanase@uaic.ro; 3 ”Anastasie Fatu” Botanical Garden, “Alexandru Ioan Cuza” University of Iasi, 7-9 Dumbrava Rosie, 700487 Iasi, Romania; 4 Faculty of Medicine and Pharmacy, “Dunarea de Jos” University, 800008 Galati, Romania; silvia.robu@ugal.ro (S.R.); denisa.batir@ugal.ro (D.B.-M.)

**Keywords:** *Dracocephalum*, antioxidant activity, Lamiaceae, flavonoids, tilianin, rosmarinic acid, medicinal plants, phenolic compounds, natural antioxidants

## Abstract

The genus *Dracocephalum* (Lamiaceae), comprising over 60 species predominantly distributed in Europe and Asia, has historically been used in traditional medicine and has recently attracted growing scientific interest due to its diverse pharmacological and phytochemical properties. Despite increasing pharmacological and phytochemical investigations, the antioxidant potential and related bioactivities of *Dracocephalum* species remain fragmented across individual studies, with limited efforts to comparatively integrate evidence on phytochemical diversity, antioxidant relevance, and pharmacological variability. Therefore, this review consolidates and critically evaluates current knowledge regarding the phytochemical diversity, antioxidant potential, and therapeutic applications of *Dracocephalum* species, emphasizing their bioactive compounds and antioxidant-driven mechanisms. Particular attention is given to polyphenolic and phenolic constituents—including flavonoids, phenolic acids, terpenoids, and volatile compounds, with rosmarinic acid, tilianin, luteolin derivatives, and apigenin derivatives identified as key contributors to biological activity. Unlike previous reviews, which primarily focused on isolated pharmacological effects or individual species, this study provides a comparative and integrative perspective by linking phytochemical composition with antioxidant-related activities and therapeutic implications across species. By synthesizing fragmented evidence and highlighting methodological advances in chromatography, metabolomics, and comparative analyses, this review identifies current knowledge gaps and outlines future perspectives for phytopharmaceutical, nutraceutical, and functional food applications.

## 1. Introduction

The genus *Dracocephalum*, part of the family Lamiaceae, includes more than 60 species, many of which have been traditionally used in various ethnomedical practices [1,2]. These species show a wide ecological range, thriving in habitats such as steppes, semi-arid regions, deserts, and high-altitude alpine environments across temperate Eurasia. A small number of narrowly endemic taxa are found in parts of North America and North Africa [2,3,4]. The geographic distribution of *Dracocephalum* ranges from temperate lowlands to challenging alpine regions, with the genus preferring temperate continental climates. These climates are characterized by marked seasonality, notable temperature fluctuations, and moderate rainfall, often concentrated during specific times of the year [5].

Owing to their distinctive floral structure, which resembles a dragon’s head, species within the genus *Dracocephalum* are commonly called “dragonhead” plants [3]. Moreover, many species in this genus have garnered significant scientific interest due to their phytochemical composition and the wide range of biological activities they exhibit [2]. In this context, several parts of the plant, including the aerial parts, leaves, flowers, and roots, have been extensively used for their medicinal properties [6]. In many regions, herbal remedies made from these plants are employed to treat digestive problems, respiratory issues, inflammatory diseases, fever, and cardiovascular conditions [6]. For instance, *Dracocephalum moldavica* L. has traditionally been consumed as an herbal infusion because of its pleasant aroma and potential health benefits [3]. Beyond their traditional uses, *Dracocephalum* species are also valued for their aromatic and culinary applications, particularly as herbal teas and functional beverages [3].

In recent decades, the genus *Dracocephalum* has garnered significant interest from the scientific community, mainly due to the discovery of a wide range of bioactive compounds [2]. Advances in modern analytical techniques have revealed that species of this genus are rich in secondary metabolites, including flavonoids, phenolic acids, terpenoids, lignans, and essential oil components [5,7]. Most of these compounds exhibit notably strong antioxidant properties [2]. For example, flavonoids such as tilianin, luteolin, and apigenin derivatives have been identified as major phytochemicals in several *Dracocephalum* species [5,7,8]. These compounds exhibit diverse pharmacological effects, including significant anti-inflammatory, cardioprotective, and neuroprotective activities [8]. Moreover, recent studies have reported the isolation of new compounds from this genus [9]. For instance, moldavica acid A, a novel salicylic acid derivative isolated from *Dracocephalum moldavica*, has been shown to modulate molecular pathways associated with cardiovascular protection [9].

The genus has been studied for a range of pharmacological effects, including antimicrobial, antiviral, anticancer, antioxidant, and anti-inflammatory activities [10,11,12,13]. Extracts and isolated compounds from various *Dracocephalum* species have shown promising biological effects in both laboratory and live models, emphasizing their potential for use in pharmaceuticals and nutraceuticals [12,13]. Recent research has also investigated innovative applications of *Dracocephalum*-derived compounds in fields like food technology, nanotechnology, and biomedical engineering [14,15]. For instance, essential oils from *Dracocephalum moldavica* have been incorporated into nanoemulsions and active food packaging systems to improve food preservation and quality [14]. Additionally, new nanomaterials derived from this plant have displayed potential therapeutic effects in biomedical applications [15]. Despite the increasing number of studies on the genus *Dracocephalum*, the available information remains fragmented across different scientific disciplines [2].

Therefore, a thorough review of the existing literature is needed to summarize current knowledge on botanical features, traditional uses, phytochemical composition, biological activities, and emerging research directions for these plants [2]. The aim of this review is to provide a comprehensive overview of the genus *Dracocephalum*, highlighting its ethnobotanical significance, phytochemical diversity, antioxidant activity, pharmacological effects, and recent research developments focused on innovative applications and biotechnological methods [2,3].

### 1.1. Botanical Characteristics and Taxonomy of Dracocephalum

The genus *Dracocephalum* encompasses a diverse array of species exhibiting unique morphological traits and ecological adaptations. These features, along with their taxonomic classification, offer a valuable framework for exploring the genus’ diversity and its significance in phytochemical and pharmacological research.

#### 1.1.1. Taxonomic Classification Within the Lamiaceae Family

The genus *Dracocephalum* belongs to the *Lamiaceae* family, one of the most diverse families of aromatic and medicinal plants, which includes numerous genera of pharmacological and culinary importance, such as Mentha, Salvia, and Thymus [2,3]. Members of this family are usually characterized by the presence of glandular trichomes responsible for the production of essential oils, as well as a rich diversity of secondary metabolites, including flavonoids, terpenoids, and phenolic acids [2].

Within the *Lamiaceae* family, the genus *Dracocephalum* is classified in the subfamily Nepetoideae and the tribe Mentheae, which includes several aromatic genera widely used in traditional medicine [5]. Phylogenetic analyses based on molecular markers, including ITS regions and chloroplastic DNA sequences, have confirmed the close evolutionary relationships between *Dracocephalum* and other genera within this group [5].

The genus includes over sixty accepted species, distributed predominantly in the temperate regions of Europe and Asia, with the greatest diversity observed in Central Asia and the mountainous areas of the Northern Hemisphere [2,6]. In recent decades, many species have attracted increasing scientific interest due to their phytochemical diversity and wide range of biological activities [2].

#### 1.1.2. Morphological Characteristics of the Genus

Species of the genus *Dracocephalum* are typically herbaceous plants that can be annual, biennial, or perennial, depending on environmental conditions [3]. These plants are commonly found in grasslands, steppes, and mountainous regions, where they have adapted to a variety of climatic conditions [6].

- Stems

The stems are generally upright and quadrangular, a notable morphological characteristic of the Lamiaceae family. This structure provides support and indicates the usual arrangement of vascular tissues [2,3].

- Leaves

The leaves are opposite and simple in form, with shapes ranging from lanceolate to ovate. They frequently contain glandular structures that produce and store essential oils and volatile compounds, which contribute to the plants’ aromatic properties and biological activities [2,3].

- Flowers and inflorescences

The flowers are among the most distinctive features of the genus, typically arranged in verticillaster inflorescences, a characteristic common to numerous Lamiaceae species [2,3]. The flower colors range from blue and violet to purple or white, thereby enhancing their ornamental and ecological functions [3].

- Dragonhead-shaped corolla

The nomenclature *Dracocephalum* derives from the Greek terms “drakon” (dragon) and “kephale” (head), referencing the bilabiate corolla that resembles a dragon’s head [3,16]. The upper lip curves over the reproductive structures, while the lower lip functions as a landing zone for pollinators, thereby facilitating plant–pollinator interactions and reproductive success [3,5].

#### 1.1.3. Geographic Distribution and Ecological Habitats

Species of the genus *Dracocephalum* are widely distributed across Europe, Central Asia, the Middle East, Siberia, and East Asia [6]. The genus exhibits particularly high diversity in Central Asia and western China, where numerous species grow naturally in mountainous and steppe environments [5].

In Europe, species such as *Dracocephalum moldavica* and *Dracocephalum ruyschiana* L. are commonly found in temperate regions and have been traditionally cultivated as medicinal and aromatic plants [3,17]. In the Middle East, *Dracocephalum kotschyi* Boiss. is an endemic species with significant ethnomedicinal importance [6].

In Siberia and East Asia, species such as *Dracocephalum tanguticum* Maxim., *Dracocephalum heterophyllum* Benth., and *Dracocephalum rupestre* Hance thrive in alpine and semi-arid environments and are known for their distinctive phytochemical profiles [18,19,20].

The ecological adaptability of *Dracocephalum* species enables them to grow in diverse habitats, including grasslands, rocky slopes, and high-altitude ecosystems, contributing to the extensive diversity observed within the genus [6].

#### 1.1.4. Species Diversity

The genus *Dracocephalum* encompasses numerous species exhibiting a broad spectrum of morphological and chemical characteristics [2]. Although over sixty species have been identified, only a limited number have been comprehensively investigated regarding their phytochemical composition and pharmacological attributes [2]. This uneven research focus has primarily concentrated on a limited number of species, notably *Dracocephalum moldavica* and *Dracocephalum kotschyi Boiss.*, leaving many species insufficiently studied. Consequently, our understanding of the genus relies predominantly on a small subset of species, underscoring the need to broaden comparative studies to include additional species.

Among the most extensively studied species are:

- *Dracocephalum moldavica*, known for its essential oil composition, phenolic compounds, and pharmacological activities [7,21,22]
- *Dracocephalum kotschyi,* rich in flavonoids and widely studied for its biological properties [23,24,25]
- *Dracocephalum heterophyllum*, investigated for phenolic alkaloids and antioxidant compounds [18,26]
- *Dracocephalum tanguticum*, characterized by flavonoids with cardioprotective effects [19,27]
- *Dracocephalum rupestre*, studied for its metabolomic profile and use in herbal tea preparations [20,28,29]
- *Dracocephalum multicaule* Montbr. & Aucher ex Benth., analyzed for variations in essential oil composition during different phenological stages [30,31]

**Figure 1:**
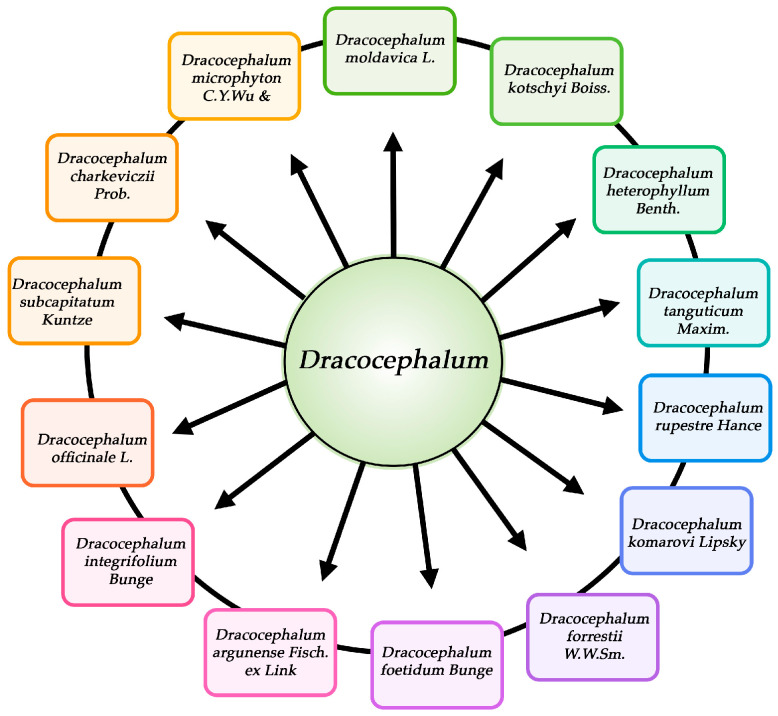
Schematic overview of representative *Dracocephalum* species discussed in the present review. The diagram summarizes the principal species covered in the literature.

The diversity of species within the genus reflects a wide range of ecological adaptations and phytochemical profiles, emphasizing *Dracocephalum* as a noteworthy subject for phytochemical and pharmacological research [2]. A simplified schematic in Figure 1 offers a visual overview of the key *Dracocephalum* species discussed in this review. It highlights the main species covered in the sections that follow.

### 1.2. Ethnobotanical and Traditional Uses

Species of the genus *Dracocephalum* have been extensively used in traditional medicine throughout Europe, Asia, and the Middle East for centuries, underscoring their enduring therapeutic and cultural significance [3,6]. Ethnobotanical research indicates that various plant components, including aerial parts, leaves, flowers, and roots, are frequently used in herbal preparations to treat a range of ailments [6].

**Figure 2:**
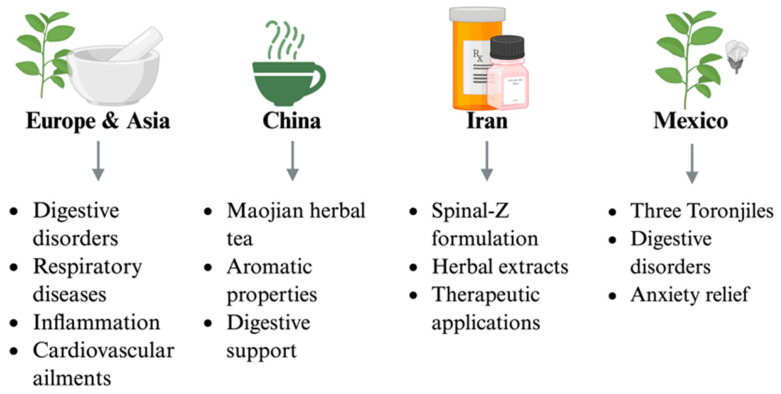
Ethnobotanical and traditional applications of *Dracocephalum* across various geographical regions. The figure consolidates its uses in Europe and Asia, including China, Iran, and Mexico, emphasizing its role in managing digestive, respiratory, inflammatory, and cardiovascular conditions. Additionally, it highlights its integration into herbal formulations, functional beverages, and traditional therapeutic preparations.

The geographical distribution and diversity of these traditional applications are summarized in Figure 2, which illustrates the use of *Dracocephalum* species across Europe, Asia, China, Iran, and Mexico. The figure emphasizes their role in addressing digestive disorders, respiratory diseases, inflammatory conditions, and cardiovascular ailments, as well as their integration into herbal formulations, functional beverages, and traditional therapeutic preparations.

#### 1.2.1. Conventional Medicinal Applications

Traditional medicinal practices have extensively used *Dracocephalum* species to treat digestive issues, respiratory ailments, inflammatory diseases, fever, and cardiovascular conditions [2,3,6].

These applications are primarily attributed to their bioactive compounds, which exhibit antioxidant, anti-inflammatory, and antimicrobial properties [2]. An illustrative example is the Iranian product Spinal-Z, which contains *Dracocephalum kotschyi*, traditionally used for cancer treatment, particularly gastrointestinal cancers [13].

Scientific investigations have identified flavonoids such as xanthomicrol as key bioactive constituents responsible for their cytotoxic and therapeutic effects [13]. Additionally, a notable ethnomedicinal example from Mexican traditional medicine is the remedy known as “three toronjiles,” a herbal preparation combining *Dracocephalum moldavica* with *Agastache mexicana* subsp. *mexicana* and *Agastache mexicana* subsp. *xolocotziana*, all belonging to the Lamiaceae family [32]. This preparation is traditionally administered as an infusion and is commonly used to manage gastrointestinal discomfort, stomach pain, colic, abdominal cramps, digestive disturbances, anxiety, and stress-related symptoms [32]. In Mexican folk medicine, the remedy is valued for its calming and gastrointestinal-relieving effects and has been associated with traditional treatment approaches for digestive and nervous system-related complaints [32]. Recent pharmacological investigations have further explored its spasmolytic activity, indicating that interactions among constituent species may influence the overall therapeutic effect and highlighting both the complexity and ethnopharmacological relevance of this traditional herbal combination [32]. Recent studies have further emphasized the importance of flavonoids, phenolic acids, and other bioactive constituents in supporting the therapeutic potential of medicinal plants and their traditional applications [33].

#### 1.2.2. Utilization in Herbal Teas and Functional Beverages

Several *Dracocephalum* species are popular in herbal teas and functional drinks for their appealing aroma and potential health benefits [3]. Among these, *Dracocephalum moldavica* stands out for its lemon-like scent and is often prepared as an herbal infusion [3]. A notable example is Maojian tea, made from *Dracocephalum rupestre* and traditionally enjoyed in China [20]. Metabolomic research has shown that this tea is rich in bioactive compounds, including flavonoids and phenolic acids, which enhance its antioxidant effects [20,28]. Detailed phytochemical investigations continue to expand knowledge regarding the diversity of flavonoids, phenolic compounds, and other secondary metabolites occurring in medicinal plant species [34].

#### 1.2.3. Culinary and Aromatic Applications

In addition to their medicinal applications, *Dracocephalum* species are employed in culinary practices and aromatherapy for their volatile compounds and essential oils [3]. These primarily consist of monoterpenes and other aromatic constituents that improve the flavor of herbal products and may contribute to food preservation [14,30,31].

#### 1.2.4. Cultural and Regional Practices

The ethnomedicinal applications of *Dracocephalum* vary by region, emphasizing local customs and biodiversity [6]. In Iran, *Dracocephalum kotschyi* is frequently used for gastrointestinal and inflammatory ailments [6], while in China and Central Asia, *Dracocephalum moldavica* is prominent in traditional remedies for cardiovascular and nervous system conditions [9]. Although these applications are significant, certain species are threatened by overharvesting and habitat destruction, thereby underscoring the necessity for sustainable practices and conservation initiatives [6].

## 2. Methodology

### 2.1. Study Design

This review aims to summarize and critically assess the existing scientific evidence concerning *Dracocephalum* species, with an emphasis on phytochemical diversity, antioxidant activity, biological significance, and emerging applications, paying particular attention to variations in antioxidant potential among different species. In addition to a narrative review, a bibliometric analysis was conducted to investigate research trends, thematic development, and the scholarly interest in *Dracocephalum*. The study was structured to include evidence on botanical and ethnobotanical backgrounds, phytochemical composition, antioxidant properties, pharmacological relevance, and recent scientific developments, providing a comprehensive overview of current knowledge on *Dracocephalum* species.

### 2.2. Literature Search Strategy and Data Sources

A thorough literature search was conducted using two major scientific databases, Scopus and PubMed, selected for their extensive coverage of biomedical, pharmacological, phytochemical, and natural product research. The search took place in April 2026, focusing on the keyword “*Dracocephalum*” across titles, abstracts, and keywords to ensure comprehensive retrieval of relevant publications. The strategy aimed to identify studies on phytochemical diversity, antioxidant activity, ethnobotanical significance, biological mechanisms, essential oils, pharmacological properties, metabolomics, and species-specific research on *Dracocephalum*. Special emphasis was placed on publications addressing antioxidant metabolites, modulation of oxidative stress, phytochemical characterization, and comparative biological evidence across species, as these topics are crucial to this review.

All document types indexed in the selected databases were included to ensure broad scientific coverage. Duplicate records were identified and removed using the Digital Object Identifier (DOI) to reduce redundancy and improve dataset consistency.

### 2.3. Data Processing and Analytical Approach

After retrieving the literature, the collected records were organized and processed to facilitate statistical evaluation and comparative interpretation of the scientific literature on *Dracocephalum* species. Duplicate publications across databases were identified and removed using the Digital Object Identifier (DOI) to improve data consistency and reduce redundancy.

The retrieved literature was then categorized and examined to support the evaluation of scientific development, thematic emphasis, and research evolution related to *Dracocephalum*, with particular focus on phytochemical composition, antioxidant activity, biological relevance, and emerging scientific applications. Records indexed in Scopus and PubMed were compared to provide a broader perspective on the diversity and orientation of scientific research involving the genus.

**Figure 3:**
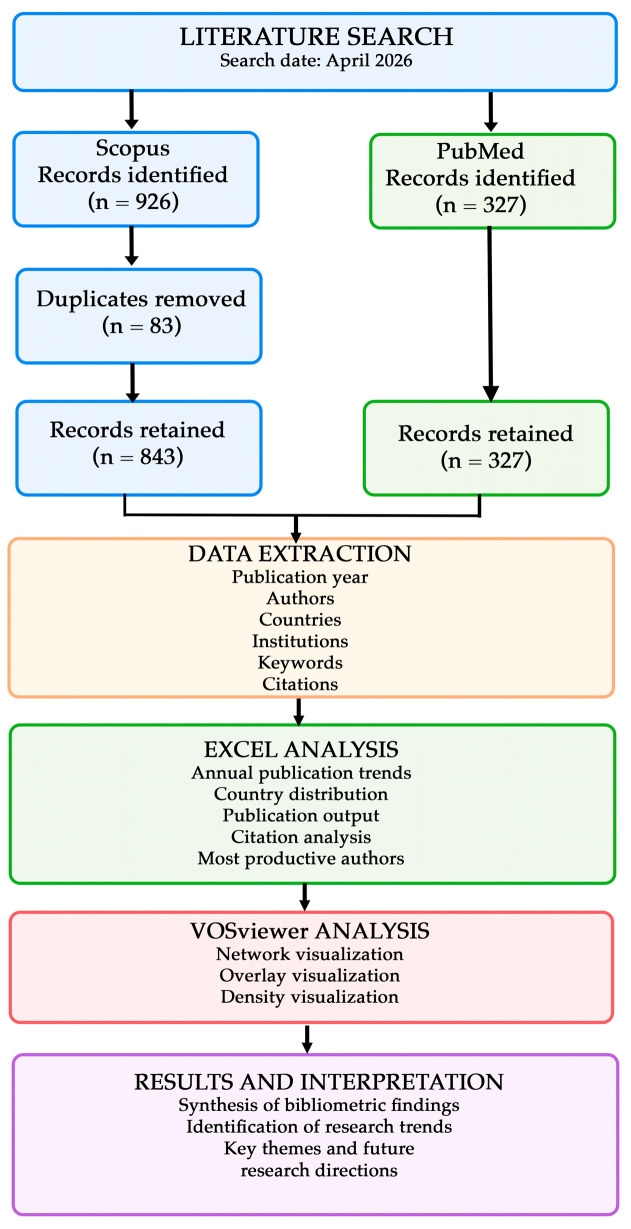
Methodological workflow of literature retrieval, data processing, and bibliometric analysis. Records were retrieved from the Scopus (*n* = 926) and PubMed (*n* = 327) databases in April 2026 using the keyword “*Dracocephalum*”. Following duplicate removal and data screening, 843 records from Scopus and 327 records from PubMed were retained for analysis. Bibliometric data were extracted and analyzed using Microsoft Excel for publication trends, country distribution, citation analysis, and research productivity assessment, while VOSviewer was employed for network, overlay, and density visualizations of keyword co-occurrence patterns and research themes.

Data organization, statistical processing, and graphical visualization were performed using Microsoft Excel. Network visualization and thematic mapping were conducted using VOSviewer (version 1.6.20; Centre for Science and Technology Studies, Leiden University, Leiden, The Netherlands), enabling comparative interpretation of scientific relationships, thematic organization, and research development patterns across the retrieved literature. A schematic overview of the study methodology is presented in Figure 3. The workflow summarizes the literature retrieval process from Scopus and PubMed, record screening and duplicate removal procedures, data extraction, and the bibliometric analyses conducted using Microsoft Excel and VOSviewer to evaluate publication trends, geographic distribution, citation patterns, and research themes.

## 3. Results and Discussion

In accordance with the specified methodological framework, the literature was meticulously reviewed to provide a comprehensive overview of current scientific knowledge on *Dracocephalum* species, with particular emphasis on antioxidant activity and its phytochemical basis. To facilitate a coherent interpretation of the literature, bibliometric findings are first presented to contextualize scientific development and research priorities, followed by phytochemical and antioxidant-related evidence. This section integrates bibliometric data, phytochemical findings, antioxidant mechanisms, and species-specific biological insights to facilitate a clear and comparative interpretation of the extant literature.

The initial section (Section 3.1) outlines bibliometric results concerning publication trends, global distribution of research activity, sources of publications, authorship patterns, thematic progression, and keyword co-occurrence analyses. Comparing data from Scopus and PubMed offers insights into how scientific interest in *Dracocephalum* has developed over time, while VOSviewer analyses highlight key thematic links and research directions. These results provide context for the field’s scientific growth and pinpoint primary research areas, especially in phytochemistry, pharmacology, and antioxidant studies.

The following sections explore the phytochemical diversity of *Dracocephalum* species and the biological significance of their major secondary metabolites, including flavonoids, phenolic acids, terpenoids, lignans, and volatile compounds. Special focus is placed on metabolites associated with antioxidant activity, including rosmarinic acid, tilianin, luteolin derivatives, apigenin derivatives, and other compounds involved in oxidative stress regulation and radical scavenging.

This review compares evidence for antioxidant activity across *Dracocephalum* species, focusing on similarities and differences in phytochemical content, antioxidant capacity, biological significance, and experimental findings. It also discusses the mechanisms underlying antioxidant effects—such as free radical scavenging, metal ion chelation, preventing lipid peroxidation, and enhancing natural antioxidant defenses—to provide a clear understanding of how these biological activities operate across various studies.

The section concludes by synthesizing current findings to highlight research gaps, methodological limitations, and future scientific directions in the pharmaceutical, nutraceutical, functional food, and biotechnology applications of *Dracocephalum*-derived antioxidant compounds.

### 3.1. Bibliometric Analysis of Dracocephalum Research

The rising scientific focus on *Dracocephalum* species highlights their complex phytochemistry, antioxidant capabilities, and wider pharmacological significance. Reviewing existing research provides valuable insight into how research priorities have evolved, the main scientific themes, and the growing interest in their biological activities and therapeutic uses.

The analysis of the retrieved literature reveals a gradual shift from descriptive botanical and taxonomic studies to research focused on phytochemistry, pharmacology, and antioxidants. There is growing interest in identifying bioactive metabolites, modulating oxidative stress, analyzing essential oil composition, and exploring new technological applications of compounds from *Dracocephalum*.

The following sections outline the main bibliometric insights on publication trends, geographic distribution of research, scientific productivity, thematic areas, and network relationships within the field. Collectively, these findings offer a broader view of the scientific progress in *Dracocephalum* research and help contextualize the increasing focus on antioxidant activity, phytochemical diversity, and species-specific biological significance.

#### 3.1.1. Temporal Trends in Scientific Publications

**Figure 4:**
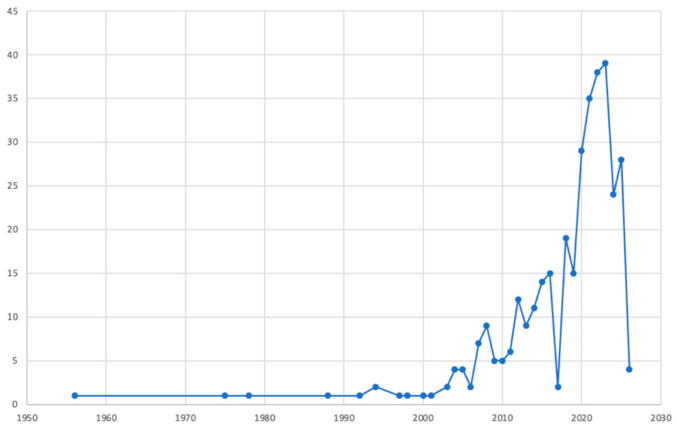
Temporal distribution of scientific publications related to the genus *Dracocephalum* indexed in the PubMed database.

**Figure 5:**
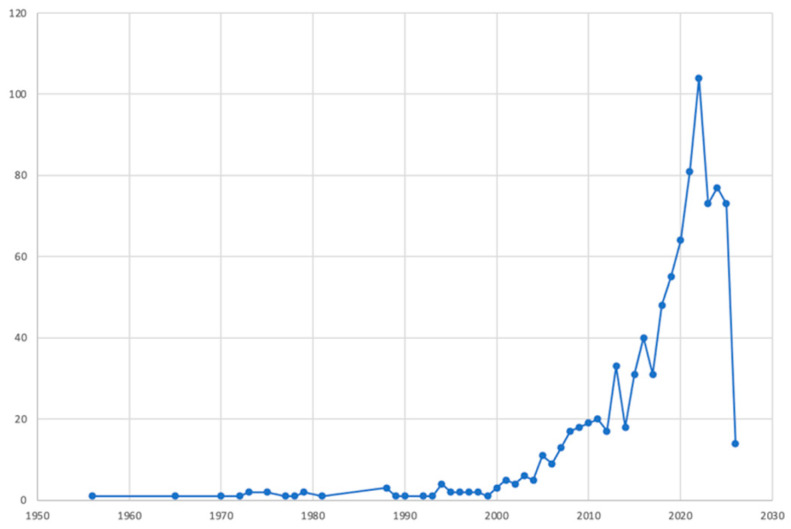
Temporal distribution of scientific publications related to the genus *Dracocephalum* indexed in the Scopus database.

Publication trends in the PubMed and Scopus databases show a consistent increase in *Dracocephalum* research, particularly after 2010, with accelerated growth in recent years (Figure 4 and Figure 5). Although publication output differs between databases, both indicate growing interest in the phytochemical composition, pharmacological properties, and antioxidant activity of *Dracocephalum* species. This increase may be associated with advances in analytical methodologies and the growing interest in plant-derived bioactive compounds [2,7].

#### 3.1.2. Geographic Distribution of Research Activity

**Figure 6:**
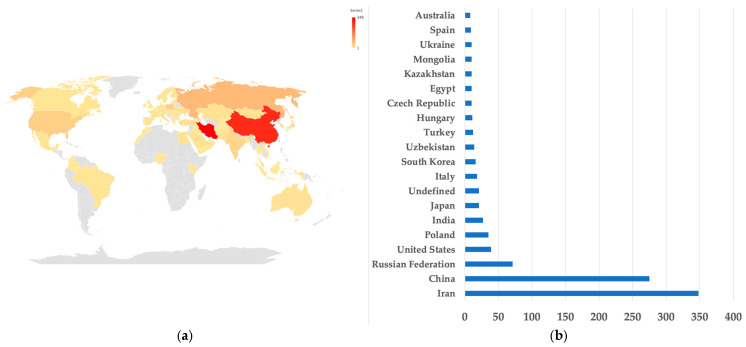
Global distribution of scientific publications related to the genus *Dracocephalum* indexed in the Scopus database. (**a**) Geographic map showing the distribution of publications across 72 countries identified through a Scopus search using the keyword “*Dracocephalum*” (April 2026 dataset); color intensity reflects publication output, ranging from countries with a single publication to the highest-producing countries, including Iran (348 publications) and China (275 publications). (**b**) Bar chart showing the top 20 countries ranked by publication output.

Research activity on *Dracocephalum* is predominantly concentrated in Asia and Europe, regions corresponding to the natural distribution and traditional medicinal use of several species within the genus (Figure 6). A bibliometric analysis of publications indexed in the Scopus database identified contributions from 72 countries based on the keyword “*Dracocephalum*” (data retrieved in April 2026 and subsequently curated to remove irrelevant records). As illustrated in Figure 6a, Iran and China are the leading contributors, with 348 and 275 publications, respectively, followed by the Russian Federation (71 publications), the United States (39 publications), Poland (35 publications), and India (27 publications). Countries with lower publication output are represented by lighter shades, whereas those with higher output are shown in darker shades. To improve readability, Figure 6b presents the top 20 countries ranked by publication output, highlighting the major geographical contributors to *Dracocephalum* research.

#### 3.1.3. Research Focus and Scientific Domains

**Figure 7:**
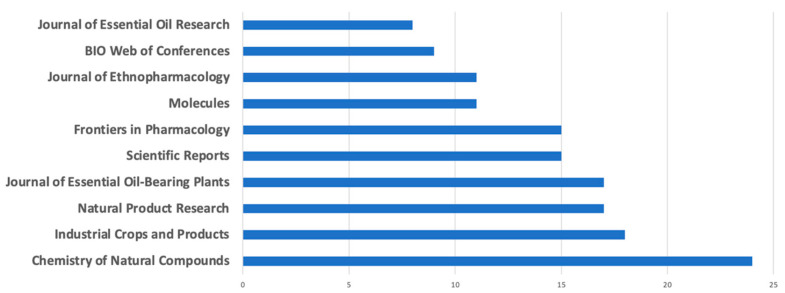
Top 10 journals publishing research on *Dracocephalum* based on Scopus data, showing the number of publications per journal.

Beyond geographical distribution, the analysis of publication sources and research themes provides additional insight into the main scientific directions of *Dracocephalum* research. The most productive journals include *Chemistry of Natural Compounds*, *Industrial Crops and Products*, and *Natural Product Research*, reflecting the field’s interdisciplinary nature, particularly in phytochemistry, pharmacology, and natural product science (Figure 7).

Research on *Dracocephalum* species primarily focuses on phytochemistry, antioxidant activity, pharmacological effects, and essential oil composition. Numerous studies investigate bioactive compounds such as flavonoids, phenolic acids, terpenoids, and rosmarinic acid, while recent research increasingly employs advanced methodologies including metabolomics, molecular docking, and nanotechnology-based approaches [28,35].

**Figure 8:**
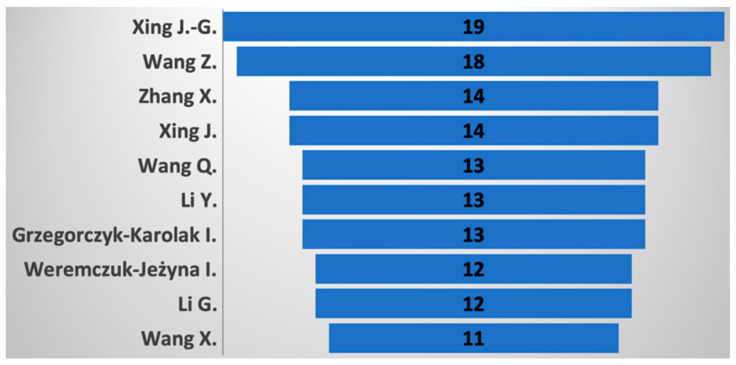
Top 10 authors publishing research on *Dracocephalum* based on Scopus data, ranked by the number of publications.

Analysis of authorship patterns indicates that research on *Dracocephalum* involves a diverse and internationally distributed group of researchers, with only a limited number of authors publishing multiple studies. Among the most productive authors are Xing J.-G. and Wang Z., together with several other contributors showing similar publication frequencies (Figure 8).

#### 3.1.4. Network Analysis of Research Themes Using VOSviewer

To further explore thematic relationships and the evolution of research interests within the field, a keyword co-occurrence analysis was conducted in VOSviewer using the Scopus dataset.

**Figure 9:**
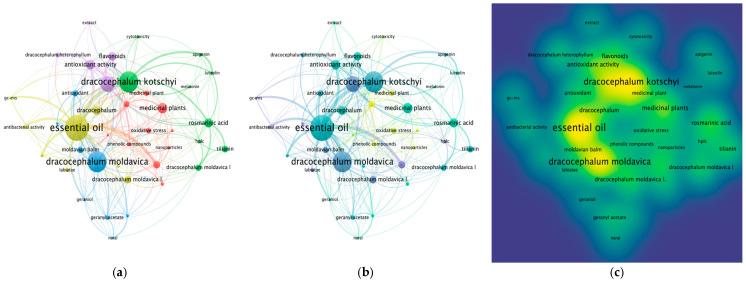
Keyword co-occurrence analysis of *Dracocephalum* research based on Scopus data using VOSviewer: (**a**) network visualization; (**b**) overlay visualization based on average publication year; and (**c**) density visualization. Node size reflects keyword frequency, while colors indicate thematic clusters, temporal evolution, and research intensity.

The analysis identified several interconnected research clusters primarily associated with essential oils, phytochemistry, pharmacological activity, and plant physiology (Figure 9a–c). Frequently occurring terms included essential oil, flavonoids, rosmarinic acid, oxidative stress, and *Dracocephalum moldavica*, highlighting the predominance of phytochemical and biological studies within the field.

The overlay visualization indicated a gradual transition from taxonomy- and biodiversity-oriented studies to molecular and pharmacological investigations, including metabolomics, gene expression, and network pharmacology. Density visualization further confirmed the central importance of essential oils and phenolic compounds in current *Dracocephalum* research. Overall, the Scopus-based analysis suggests an increasing shift toward integrative biological and pharmacological approaches.

#### 3.1.5. Comparative Analysis of Research Themes: PubMed vs. Scopus

**Figure 10:**
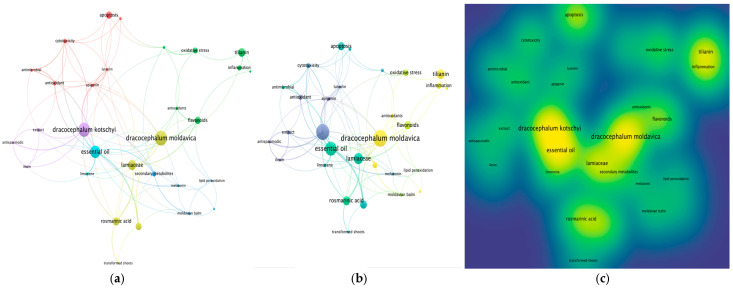
Comparative VOSviewer analysis of PubMed-indexed *Dracocephalum* research: (**a**) network visualization of keyword co-occurrence; (**b**) overlay visualization based on average publication year; and (**c**) density visualization highlighting the most intensively studied biomedical topics.

A comparative analysis of the PubMed dataset revealed a more compact and biomedical-oriented research structure than that observed in Scopus (Figure 10a–c). Frequently occurring terms included *Dracocephalum moldavica*, *Dracocephalum kotschyi*, flavonoids, rosmarinic acid, oxidative stress, inflammation, and essential oil, indicating a stronger emphasis on biological activity and therapeutic applications.

In contrast to the broader and more interdisciplinary Scopus network, themes related to taxonomy, ethnobotany, and plant physiology were less represented in PubMed, reflecting the predominantly biomedical orientation of this database. Overlay visualization indicated that PubMed-indexed studies are mainly associated with recent pharmacological and bioactivity-oriented research directions, while density visualization highlighted the central importance of essential oils, flavonoids, and oxidative stress-related studies.

Overall, the comparison demonstrates the complementary nature of the two databases: Scopus provides a broader multidisciplinary perspective, whereas PubMed captures the biomedical dimension of *Dracocephalum* research more specifically.

The bibliometric findings highlight phytochemistry and antioxidant activity as dominant research themes, thereby supporting a more detailed discussion of the phytochemical basis underlying the biological relevance of *Dracocephalum* species.

### 3.2. Phytochemical Composition of Dracocephalum and Its Relevance to Antioxidant Activity

**Figure 11:**
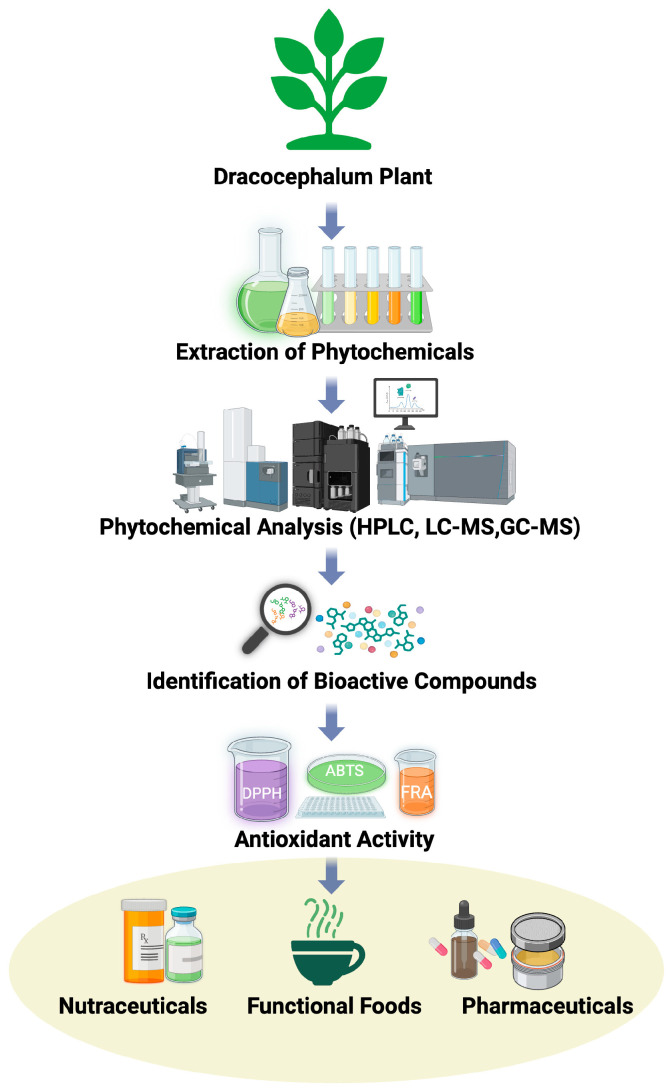
Schematic overview of the workflow for extracting, analyzing, and assessing *Dracocephalum* phytochemicals and their antioxidant activity.

Following the bibliometric analysis, the identified research trends indicate that phytochemistry and antioxidant activity represent the central scientific focus of *Dracocephalum* studies. Species of the genus *Dracocephalum* are recognized for their remarkable chemical diversity and the presence of secondary metabolites with potential biological activities, especially antioxidant properties [2,36,37]. As shown in Figure 11, the study of these bioactive compounds involves several steps, including extraction, advanced phytochemical analysis (e.g., HPLC, LC-MS, GC-MS, and NMR), identification of key constituents, and subsequent evaluation of their antioxidant activity [38].

Phytochemical studies have shown that these plants contain a wide range of bioactive compounds, including flavonoids, phenolic acids, terpenoids, lignans, alkaloids, and volatile constituents of essential oils [2,7,39,40]. They may exhibit strong antioxidant activity through mechanisms such as free radical scavenging, metal ion chelation, and inhibition of oxidative processes, with phenolic compounds and flavonoids playing a major role [21,23,41,42]. Modern analytical methods have enabled the identification of numerous compounds and the detailed profiling of phytochemicals, aiding in the discovery of new antioxidant molecules [7,9]. *Dracocephalum moldavica* has been reported to contain at least 68 compounds, including flavonoids (tilianin, luteolin and apigenin derivatives), phenolic acids (rosmarinic acid, caffeic acid), and volatile terpenoids, many of which contribute to its antioxidant activity [7,20].

The chemical makeup of *Dracocephalum* species can vary greatly depending on factors such as geographical origin, environmental conditions, developmental stage, and extraction techniques, all of which can influence both the concentration and diversity of compounds [21,30]. These variations can also significantly affect the antioxidant potential of extracts from different species.

#### 3.2.1. Volatile Compounds and Essential Oils

Among the diverse classes of secondary metabolites identified in *Dracocephalum* species, volatile compounds and essential oils represent an important group contributing to biological activity and antioxidant potential. Essential oils are among the most significant phytochemicals present in various *Dracocephalum* species, contributing to their distinctive aroma and diverse biological activities [30,43]. These volatile compounds primarily consist of terpenes, including monoterpenes and sesquiterpenes, along with their oxygenated derivatives, thereby contributing to the chemical diversity and functional attributes of the genus [30,31,44]. They are also essential for plant defense, ecological interactions, and adaptation to environmental stressors [31]. Biosynthetically, terpenes originate from isoprene units and are classified into groups such as monoterpenes (C10) and sesquiterpenes (C15), which represent the predominant constituents in *Dracocephalum* essential oils [30,45]. In addition to their antimicrobial and aromatic properties, numerous volatile constituents in *Dracocephalum* essential oils exhibit antioxidant activity by scavenging free radicals, inhibiting lipid peroxidation, and modulating oxidative processes in biological systems [30,31,46,47]. Synergistic interactions among these constituents may further augment the overall antioxidant capacity [48,49].

The composition and yield of these oils are substantially affected by factors such as the plant’s growth stage, environmental conditions, geographical origin, and processing methods [30,50,51,52]. For instance, harvesting at specific phenological stages, such as during flowering, can markedly improve both the quality and quantity of the oils [30,52]. Cultivation practices and extraction strategies also influence the accumulation of volatile compounds and their biological activities [50,51]. Concerning post-harvest processing techniques—including drying and extraction methods—these significantly affect volatile compound profiles and, consequently, antioxidant potential [53,54,55]. Modern extraction techniques, such as microwave-assisted methods, can enhance extraction efficiency and preserve heat-sensitive constituents [55,56]. Among the *Dracocephalum* species, *Dracocephalum moldavica* has been extensively investigated for its essential oil composition and biological effects. Its oil contains a complex mixture of volatile compounds and demonstrates notable antioxidant and antimicrobial activities, underscoring the importance of these constituents for plant physiology and potential industrial applications [46,57,58,59,60].

Recent investigations have also explored innovative applications, such as incorporating *Dracocephalum moldavica* essential oil into nanoemulsions and edible coatings—biodegradable films derived from seed mucilage—to enhance antioxidant and antimicrobial effects, thereby helping to preserve foods and extend their shelf life [14,61,62,63,64]. Such strategies offer promising alternatives to synthetic preservatives.

Essential oils are increasingly recognized for their multifunctional biological activities, including antioxidant, antimicrobial, and anti-inflammatory effects, rendering them valuable in pharmaceutical, cosmetic, and food industries [48,65,66,67]. Their antioxidant properties are closely linked to their chemical composition, particularly oxygenated terpenes and other bioactive compounds [46,47]. Research on other species, such as *Dracocephalum foetidum* Bunge, reveals the presence of monoterpene glycosides and phenylpropanoid derivatives, which may contribute to plant defense mechanisms and antioxidant functions [68]. Overall, volatile compounds and essential oils derived from *Dracocephalum* species constitute complex, biologically active phytochemicals influenced by environmental, genetic, and technological factors. They play a crucial role, directly or indirectly, in the genus’ antioxidant properties, emphasizing their significance for scientific research and industrial applications.

#### 3.2.2. Flavonoids

Flavonoids are among the most abundant and biologically significant secondary metabolites in *Dracocephalum* species, contributing substantially to their medicinal properties, particularly their antioxidant activity [7,69,70,71]. These compounds are recognized as effective natural antioxidants, capable of donating hydrogen atoms or electrons, thereby neutralizing reactive oxygen species (ROS) and preventing oxidative chain reactions in biological systems [7,72,73].

**Figure 12:**
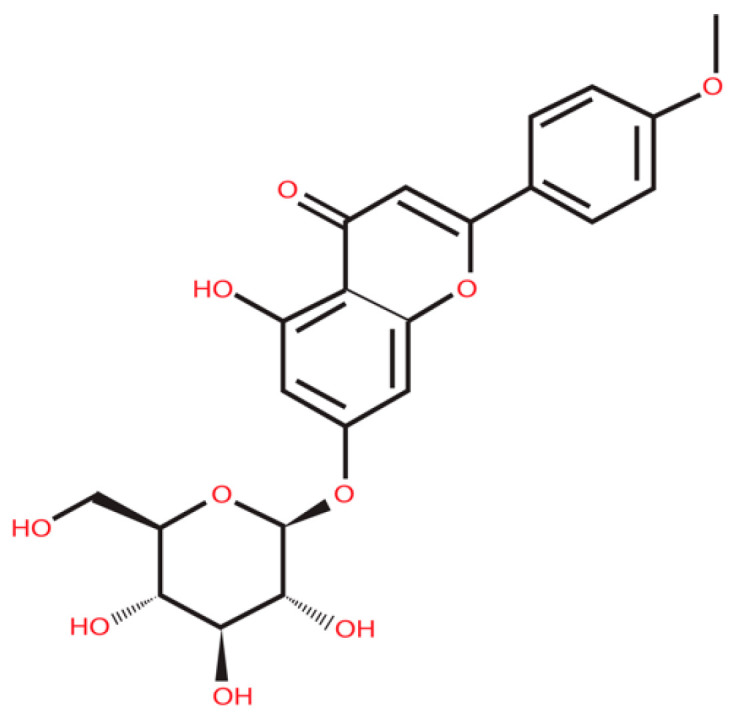
Chemical structure of tilianin (acacetin-7-O-β-D-glucopyranoside) isolated from *Dracocephalum* species.

Structurally, flavonoids possess a C6-C3-C6 structure (two aromatic rings linked by a 3-carbon heterocyclic unit) and are classified into various subclasses—flavones, flavonols, flavanones, isoflavones, anthocyanidins, and flavanols—which differ according to substitution patterns and oxidation levels [74,75]. Flavones, such as tilianin, luteolin derivatives, apigenin derivatives, and acacetin glycosides, are the most commonly reported in *Dracocephalum* [7,8,76,77]. Tilianin (acacetin-7-O-β-D-glucopyranoside), the main flavonoid in *Dracocephalum moldavica*, has attracted considerable attention due to its potent antioxidant and cardioprotective effects, establishing it as a marker compound for the genus (see Figure 12) [8,78,79,80]. Its antioxidant activity is associated with its ability to regulate oxidative stress pathways, reduce ROS production, and protect cellular structures from damage [72,78].

Advanced analytical techniques, such as UPLC-MS and LC-MS, have facilitated the identification of numerous flavonoids in *Dracocephalum*, including aglycones and glycosylated forms [76,81,82]. These methodologies have broadened the understanding of the diversity and distribution of flavonoids across different plant parts and species [81,83,84].

In *Dracocephalum moldavica*, flavonoids are key bioactive constituents, and their levels are strongly correlated with antioxidant activity, as demonstrated by various laboratory assays [70,76,85,86]. The increased flavonoid content generally corresponds to increased radical-scavenging capacity, thus underscoring their role in the plant’s antioxidant potential [70,72].

Studies on *Dracocephalum kotschyi* have identified methoxylated flavones, including xanthomicrol, luteolin, and apigenin derivatives, which are integral to the plant’s biological activity [13,74]. In particular, xanthomicrol has been highlighted as a major bioactive compound with substantial pharmacological potential, including antioxidant and cytotoxic properties [13].

Techniques such as two-dimensional liquid chromatography combined with HPLC-DPPH assays have allowed the isolation of antioxidant flavonoids from *Dracocephalum heterophyllum*, confirming their direct involvement in radical scavenging [26]. DPPH-guided fractionation further confirms the significant contribution of these flavonoids to the antioxidant effects observed in plant extracts [26].

Flavonoid profiles and accumulation levels can vary considerably due to environmental factors, cultivation conditions, and developmental stages, which influence the antioxidant efficacy of extracts [84,87,88].

Flavonoids from *Dracocephalum rupestre* and related plants also demonstrate notable antioxidant activity, supporting their traditional use as functional foods and herbal remedies [89,90]. The presence of flavonoid glycosides and phenylpropanoid derivatives further enhances their biological significance [91].

Overall, flavonoids constitute a fundamental component of the phytochemical composition of *Dracocephalum* and are vital for its antioxidant properties. Their structural diversity and ability to modulate oxidative stress highlight their importance as bioactive agents with potential applications in pharmaceuticals, nutraceuticals, and functional foods [7,72,92].

#### 3.2.3. Phenolic Acids

Alongside flavonoids, phenolic acids constitute another major group of antioxidant-related phytochemicals contributing to the biological relevance of *Dracocephalum* species. Phenolic acids are found widely in *Dracocephalum* species, significantly contributing to their antioxidant and overall pharmacological effects [7,93,94]. These compounds have one or more hydroxyl groups attached to aromatic rings, which confer strong redox activity, enabling them to act as effective antioxidants in biological systems [93,95].

The antioxidant activity of phenolic acids mainly comes from their ability to donate hydrogen atoms or electrons, scavenge reactive oxygen species (ROS), chelate transition metals, and inhibit lipid peroxidation [95,96,97]. Additionally, phenolic acids can influence cellular signaling pathways involved in oxidative stress and inflammation, thereby providing cellular protection [98,99,100].

Among the phenolic acids identified in *Dracocephalum*, rosmarinic acid is distinguished as particularly prominent and extensively studied. It is present in several species and is recognized for its potent radical-scavenging capability and ability to inhibit oxidative chain reactions [7,101,102]. The antioxidant efficacy of rosmarinic acid is associated with its chemical structure, notably its multiple hydroxyl groups that stabilize free radicals and enhance its redox potential [102,103,104]. Furthermore, rosmarinic acid has demonstrated supplementary health benefits, including anti-inflammatory and cardioprotective properties, attributable to its antioxidant activity [100,103].

Various *Dracocephalum* species also contain phenolic acids, including caffeic acid and cinnamic acid derivatives [9]. These compounds support the antioxidant properties of plant extracts by protecting lipids, proteins, and nucleic acids from oxidative damage and enhancing the body’s natural antioxidant defenses [9,97,99]. The biological effects of phenolic acids rely heavily on their chemical structure—particularly the number and position of hydroxyl groups, the degree of conjugation, and the presence of substituents—which influence their ability to scavenge radicals and chelate metals [95,105]. Structure–activity studies show that phenolic acids with ortho-dihydroxyl groups have stronger antioxidant effects due to the increased stability of the phenoxyl radicals they produce [95]. In addition to direct radical scavenging, phenolic acids can regulate gene expression, enzyme activity, and cellular signaling pathways associated with oxidative stress, thereby exerting indirect antioxidant effects [99,100]. Their metabolites might also contribute to biological activity, indicating that both parent compounds and their transformation products are involved in antioxidant defense [99].

The variety of phenolic compounds in *Dracocephalum* suggests potential synergistic interactions with flavonoids and other polyphenols, which could enhance the overall antioxidant capacity of the extracts [93,106]. This synergy is especially relevant in complex plant matrices where multiple compounds work together more effectively to control oxidative processes than individual components [94,106].

Recent phytochemical investigations have identified novel phenolic derivatives, including moldavica acid A, a new salicylic acid derivative isolated from *Dracocephalum moldavica* [9]. This compound influences pathways associated with cardiovascular health, thereby linking antioxidant activity to cardiovascular benefits [9]. Progress in analytical methodologies such as HPLC, LC–MS, and UPLC has significantly enhanced the detection, quantification, and analysis of phenolic acids in *Dracocephalum* species [107,108], facilitating comprehensive profiling and a deeper understanding of the correlation between chemical composition and antioxidant activity [96,109].

Extraction methods and processing conditions are pivotal in determining the yield and composition of phenolic acids, thereby influencing their biological activity and potential applications [109,110,111]. Consequently, optimizing extraction techniques is imperative to enhance the recovery of antioxidant compounds from plants [96,111].

Overall, phenolic acids are a fundamental component of the phytochemical profile of *Dracocephalum* species and are integral to their antioxidant effects through multiple mechanisms. Their structural diversity, potent redox properties, and capacity to act synergistically with other bioactive compounds underscore their importance as primary contributors to the genus’ pharmacological, nutraceutical, and functional attributes [7,93,94].

#### 3.2.4. Other Secondary Metabolites

*Dracocephalum* species encompass a range of secondary metabolites extending beyond flavonoids, phenolic acids, and volatile terpenoids. These include lignans, alkaloids, phenylpropanoids, and other phenolic derivatives, thereby augmenting the chemical and biological diversity of the genus [7,18,112]. Although less extensively studied, these compounds are likely to contribute to antioxidant effects through diverse and synergistic mechanisms [7,93]. Lignans, particularly in *Dracocephalum moldavica*, represent a noteworthy category, encompassing over 28 distinct variants, including newly identified dracomolphins A–E, which have been isolated for their therapeutic potential. These phenolic constituents are associated with the plant’s antioxidant, anti-inflammatory, and antitumor properties and frequently function as Nrf2 transcriptional activators [112].

Recent research indicates the presence of numerous structurally diverse compounds exhibiting antioxidant, anti-inflammatory, and cytoprotective activities, thereby implying their broader biological significance [113,114,115]. Certain secondary metabolites may collaborate synergistically with flavonoids and phenolic acids, enhancing antioxidant capacity through their combined effects [93,106].

Advanced techniques such as LC–MS and UPLC have facilitated detailed identification of these minor compounds, revealing new bioactive substances [35,107,108], thereby enhancing our understanding of their distribution and potential pharmacological applications [35,116]. Additionally, iridoid glycosides such as harpagide and harpagide acetate have been identified in *Dracocephalum moldavica*, further enhancing its chemical diversity and biological activity [117,118].

In summary, these secondary metabolites enhance the chemical diversity of *Dracocephalum*, are important for antioxidant activity, and deserve further investigation in phytochemistry and pharmacology [7,37,113,114,115,116].

### 3.3. Antioxidant Activity of Dracocephalum Species

Oxidative stress plays a major role in the development of many chronic diseases, including cardiovascular disorders, neurodegenerative diseases, metabolic syndrome, and cancer. It results from an imbalance between reactive oxygen species (ROS) production and the ability of biological systems to neutralize these reactive intermediates with endogenous antioxidants. Consequently, natural antioxidants from medicinal plants have gained attention for their potential to prevent oxidative damage and maintain cellular homeostasis [2].

The genus *Dracocephalum* has been extensively studied for its rich content of natural antioxidant compounds. Many species contain high levels of phenolic acids, flavonoids, terpenoids, and other secondary metabolites with potent radical-scavenging abilities, thereby enhancing their pharmacological effects [2,7]. Notably, flavonoids such as tilianin, luteolin derivatives, apigenin derivatives, and xanthomicrol, along with phenolic acids like rosmarinic acid and caffeic acid derivatives, have been identified as key antioxidant components in various *Dracocephalum* species [2,7]. To better contextualize antioxidant-related evidence across the genus, the following sections provide comparative summaries of species-specific findings on phytochemical composition, antioxidant potential, and reported biological relevance.

#### 3.3.1. *Dracocephalum moldavica* L.

Among the species of the genus, *Dracocephalum moldavica* has received particular attention due to its rich phytochemical composition and strong antioxidant capacity [119]. A thorough investigation of polyphenolic extracts from *Dracocephalum moldavica* showed significant antioxidant, anti-inflammatory, and antigenotoxic effects [120]. The authors observed that the extract’s antioxidant activity was closely related to its high levels of phenolic compounds, particularly flavonoids and phenolic acids, which are known to effectively scavenge free radicals and chelate metals [41]. The study indicated that these compounds could help protect against oxidative stress-related cellular damage [41,121]. Similarly, research on extracts from *Dracocephalum moldavica* seeds prepared with different extraction methods revealed considerable antioxidant activity. Researchers found that extraction techniques significantly affect both phenolic content and antioxidant potential, with polar solvents generally yielding higher phenolic levels and stronger radical-scavenging effects [21,122,123]. Advanced analytical techniques, such as UPLC-Q-TOF-MS/MS, have enabled the identification of numerous antioxidant compounds in *Dracocephalum moldavica*. These analyses identified a wide variety of flavonoids and phenylpropanoids, including derivatives of luteolin-7-O-glucuronide, apigenin-7-O-glucuronide, rosmarinic acid, tilianin, and acacetin, all of which are known for their potent antioxidant properties [7,120,124]. In addition to in vitro assays, several studies have explored the biological significance of these antioxidant compounds. For instance, research on *Dracocephalum moldavica* tea shows that the plant may offer beneficial metabolic effects, partly due to its antioxidant properties. The study showed that drinking *Dracocephalum moldavica* tea alleviated metabolic issues caused by a high-fat diet, suggesting that its bioactive compounds might help reduce oxidative stress and lipid metabolism problems [92,125].

Recent investigations have also explored the utilization of *Dracocephalum moldavica* seed mucilage in the development of biodegradable edible films. These films, derived from seed mucilage, demonstrate promising physicochemical properties and possess potential applications within the food industry, including the incorporation of antioxidant compounds [126]. The bioactive constituents in the mucilage matrix may enhance the films’ antioxidant capacity, suggesting their potential as natural packaging solutions that mitigate oxidative spoilage in food products [126]. Furthermore, these biodegradable films provide an environmentally sustainable alternative to synthetic packaging, combining antioxidant functionalities with principles of sustainable development [126]. This underscores the versatility and significance of *Dracocephalum moldavica* within the food and nutraceutical sectors [126].

In addition to *Dracocephalum moldavica*, several other *Dracocephalum* species have been investigated for their antioxidant-related phytochemicals and biological activity, highlighting both shared characteristics and species-specific differences in phytochemical composition and antioxidant potential.

#### 3.3.2. *Dracocephalum kotschyi* Boiss.

**Figure 13:**
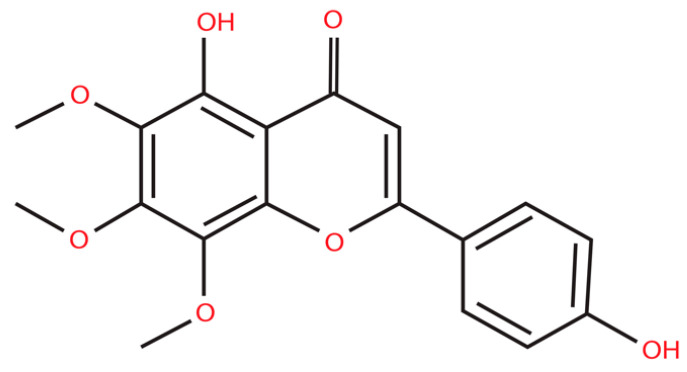
Chemical structure of xanthomicrol, a major flavonoid with antioxidant activity isolated from *Dracocephalum kotschyi*.

*Dracocephalum kotschyi*, a species with a long history in traditional Iranian medicine, has been extensively studied for its antioxidant properties. Research shows that extracts from its aerial parts have strong antioxidant activity, mainly due to high levels of flavonoids and phenolic compounds [23,127,128,129]. Notably, xanthomicrol, luteolin, and apigenin derivatives are key contributors to this activity [13]. The main bioactive compound appears to be a flavone called xanthomicrol (Figure 13), which can inhibit the growth of various cancer cells more selectively than doxorubicin. This compound has been identified as a key bioactive component responsible for several pharmacological effects of the species [13]. Environmental factors and cultivation methods also impact the production of antioxidants in this plant [130]. Studies indicate that elicitors or nanoparticles can boost secondary metabolite production and enhance antioxidant enzyme activity, suggesting that biotechnological strategies could improve their antioxidant potential [24,33,131,132].

Recent studies further substantiate the considerable antioxidant and biological potential of *Dracocephalum kotschyi*. Analyses of essential oil composition have identified a complex profile enriched with bioactive compounds that exhibit demonstrable antioxidant, antimicrobial, and cytotoxic activities, thereby underscoring its pharmacological significance [133,134,135]. Comparative investigations between wild and cultivated specimens have indicated that environmental conditions markedly influence essential oil composition and antioxidant capacity, underscoring the significance of ecological factors in metabolite variability [136,137,138,139,140,141]. Furthermore, drying methods have been shown to substantially affect phytochemical content and antioxidant properties, implying that post-harvest processing is instrumental in maintaining bioactivity [142].

Phytochemical investigations have identified several key flavonoids, including calycopterin and luteolin derivatives, that contribute to the species’ antioxidant and anti-inflammatory activities [143,144]. These compounds are also associated with immunomodulatory effects, as demonstrated by studies showing the regulation of inflammatory mediators and transcription factors in activated macrophages [145]. Moreover, recent research has highlighted the inhibitory effects of *Dracocephalum kotschyi* extracts on enzymes implicated in metabolic disorders, such as type 2 diabetes, further supporting its therapeutic potential [146,147].

In addition to antioxidant activity, *Dracocephalum kotschyi* demonstrates a broad spectrum of biological effects, including anti-inflammatory, antimicrobial, antiparasitic, and anticancer properties. Experimental research has confirmed its efficacy in mitigating inflammation in animal models, inhibiting the proliferation of pathogenic microorganisms, and inducing apoptosis in cancer cell lines [135,148,149,150,151,152,153,154]. Moreover, essential oils and plant extracts have exhibited activity against Toxoplasma gondii, suggesting potential antiparasitic applications.

Biotechnological approaches have also been explored to enhance the production of valuable secondary metabolites. Hairy root cultures have been identified as a promising source of rosmarinic acid and flavonoids, offering a controlled system for increasing the yield of antioxidant compounds [155]. Additionally, nanotechnology-based studies, including the synthesis of gold and silver nanoparticles using plant extracts, have demonstrated enhanced biological activities and potential applications in medicine and biotechnology [156,157,158,159,160].

In addition to pharmacological research, recent investigations have explored innovative applications of *Dracocephalum kotschyi* essential oil in food preservation. For instance, emulsion-based edible films incorporating this essential oil into chitosan–gelatin composites have demonstrated enhanced antioxidant and antimicrobial properties, thereby contributing to the preservation of grape quality during storage [61]. These findings underscore not only the antioxidant efficacy of *Dracocephalum kotschyi* but also its potential role in functional food systems and natural preservation technologies, where plant-derived antioxidants are crucial for extending shelf life and maintaining product quality [61,161].

#### 3.3.3. *Dracocephalum heterophyllum* Benth.

The antioxidant capacity of *Dracocephalum heterophyllum* has been extensively investigated using advanced chromatographic and analytical techniques, thereby enabling the identification of several bioactive compounds with significant radical-scavenging activity. A notable study employed preparative two-dimensional liquid chromatography, combined with an online HPLC-DPPH assay, to successfully isolate multiple antioxidant constituents from the plant’s aerial parts [26]. These findings provide strong evidence that *Dracocephalum heterophyllum* represents a valuable natural source of antioxidant compounds.

In addition to flavonoids, phenolic alkaloids isolated from this species have also demonstrated antioxidant activity, further highlighting its chemical diversity and pharmacological potential [18]. These compounds may contribute to antioxidant effects through both direct radical scavenging mechanisms and modulation of oxidative stress-related pathways [99].

Furthermore, the use of advanced analytical and bioactivity-guided fractionation techniques has enabled a more accurate correlation between chemical composition and antioxidant activity in *Dracocephalum heterophyllum*. This comprehensive approach demonstrates that the species encompasses multiple categories of bioactive compounds that function synergistically to augment its antioxidant capacity [26,162].

Recent studies have expanded the understanding of the antioxidant and pharmacological properties of *Dracocephalum heterophyllum*. Extracts rich in phenylethanoids and flavonoids have demonstrated strong antioxidant activity, confirming their ability to neutralize free radicals and reduce oxidative stress [163]. Additionally, ursolic acid-rich extracts have exhibited significant antioxidant, antidiabetic, and cytotoxic activities, suggesting a multifunctional therapeutic potential [164].

The antioxidant properties of *Dracocephalum heterophyllum* are closely linked to its protective function across diverse disease models. Empirical research has demonstrated hepatoprotective effects, wherein plant extracts mitigate oxidative damage and inflammation in models of acute hepatitis [165,166]. Likewise, extracts have been documented to alleviate nonalcoholic steatohepatitis (NASH), recently also known as metabolic dysfunction-associated steatohepatitis (MASH) and fibrosis by modulating oxidative stress, bile acid metabolism, and inflammatory responses [167,168].

Additionally, several isolated compounds, such as furanocoumarins and samwinol, have been recognized as potent antioxidants with neuroprotective effects. These substances can mitigate oxidative stress-induced damage and reduce neuroinflammation, highlighting their potential in neurodegenerative disease research [169,170]. Beyond its antioxidant capabilities, *Dracocephalum heterophyllum* exhibits various biological activities, including anti-inflammatory, antimicrobial, antidiabetic, and cardioprotective effects. Its essential oils and extracts have shown antimicrobial and antidiabetic properties, underscoring their pharmacological importance [171,172]. Moreover, flavonoid fractions have been found to protect hypertrophic cardiomyocytes, suggesting benefits for cardiovascular health [173].

These findings collectively suggest that *Dracocephalum heterophyllum* is a rich source of bioactive compounds with strong antioxidant properties and multiple therapeutic uses. Its diverse phytochemical profile and wide range of biological activities make it a promising candidate for further pharmacological and biotechnological research.

#### 3.3.4. *Dracocephalum tanguticum* Maxim.

*Dracocephalum tanguticum* has garnered significant scientific attention due to its rich phytochemical composition and extensive biological activities, notably its antioxidant and protective effects. Extensively used in traditional Tibetan medicine, this species contains a variety of bioactive compounds, including flavonoids, triterpenoids, sesquiterpenes, and phenolic derivatives, each contributing to its pharmacological properties [174,175,176,177,178].

Flavonoids extracted from *Dracocephalum tanguticum* demonstrate considerable antioxidant activity, which is intricately linked to their capacity to reduce oxidative stress. Research indicates that these compounds primarily protect cardiomyocytes from doxorubicin toxicity by diminishing reactive oxygen species and stabilizing cell membranes, thereby promoting cell survival and averting oxidative damage [19]. In addition to their antioxidant properties, certain glucoside derivatives can induce vasorelaxation through nitric oxide pathways, underscoring the connection between antioxidant effects, endothelial function, and cardiovascular health [27]. Consequently, these flavonoids provide both antioxidative and cardioprotective benefits.

Recent studies have advanced our understanding of the pharmacological effects of *Dracocephalum tanguticum*, particularly its protective properties in the liver, nervous system, inflammation, and cancer. Both in vivo and in vitro investigations demonstrate that the plant’s ethyl acetate extract can mitigate alcohol-induced liver damage by activating the Nrf2/Keap1 pathway and suppressing NF-κB-mediated inflammation [174]. These findings underscore the significance of augmenting antioxidant defenses as a fundamental mechanism underlying the plant’s hepatoprotective effects.

Transcriptomic analyses at the molecular level have elucidated the biosynthesis of phenolic compounds, especially rosmarinic acid, a well-known antioxidant. Several genes involved in the biosynthetic pathway of rosmarinic acid have been identified, demonstrating the plant’s capacity to produce substantial levels of this bioactive compound across various organs [175]. These results highlight *Dracocephalum tanguticum* as a valuable natural source of antioxidants with promising medicinal and biotechnological uses.

The antioxidant properties of *Dracocephalum tanguticum* are associated with significant neuroprotective advantages. Studies demonstrate that flavonoid-rich extracts enhance antioxidant enzyme activity, increase neurotrophic factors, and reduce oxidative damage in models of cerebral ischemia, thereby protecting neurons from oxidative stress and promoting neurological health [176,177].

In addition to antioxidant compounds, recently identified sesquiterpenes from this species exhibit potent anti-inflammatory effects, suggesting their potential cooperative role in modulating oxidative and inflammatory responses [178]. Furthermore, extracts of *Dracocephalum tanguticum* have demonstrated antitumor and antiproliferative properties in oncological models, including glioblastoma cells, by inducing apoptosis through the regulation of pathways associated with oxidative stress, cell death, and cellular survival [158,179,180].

*Dracocephalum tanguticum* is recognized as a valuable medicinal plant renowned for its antioxidant, anti-inflammatory, hepatoprotective, neuroprotective, cardioprotective, and cytoprotective properties. Its extensive phytochemical composition, corroborated by ongoing molecular and pharmacological research, highlights its potential in the development of natural therapeutic agents [19,27,158,174,175,176,177,178,179,180]. Nevertheless, further investigations are necessary to comprehensively elucidate its mechanisms and to facilitate the effective translation of these findings into clinical applications.

#### 3.3.5. *Dracocephalum rupestre* Hance

*Dracocephalum rupestre* is widely used as Maojian herbal tea in China and has long been esteemed for its health-enhancing properties. Recent metabolomic and phytochemical investigations have yielded comprehensive insights into its chemical profile, demonstrating that the plant is abundant in phenolic compounds and flavonoids, which are principal contributors to its antioxidant efficacy [20,28].

The antioxidant properties of *Dracocephalum rupestre* are linked to its capacity to neutralize free radicals, inhibit oxidative processes, and safeguard biological molecules from oxidative harm. Furthermore, investigations into its volatile and aroma-active compounds have demonstrated that these constituents not only enhance the distinctive flavor of Maojian tea but may also contribute to its biological effects [29,35,181].

Furthermore, specific flavonoids identified in this species, such as eriodictyol, have been reported to exhibit significant antioxidant and neuroprotective effects. Eriodictyol has been shown to modulate oxidative stress pathways and reduce reactive oxygen species production, thereby contributing to cellular protection and potential therapeutic effects [182,183,184,185].

In addition to these findings, experimental research has demonstrated that extracts of *Dracocephalum rupestre* possess significant hepatoprotective effects. In vivo studies have shown that the plant extract can mitigate carbon tetrachloride (CCl_4_)—induced liver damage by enhancing antioxidant enzyme activity and reducing oxidative stress markers, thereby protecting hepatic tissue from injury [89]. These advantageous effects are primarily attributable to its high content of phenolic compounds and flavonoids, which play a key role in attenuating oxidative damage and inflammation. Furthermore, the combined antioxidant and hepatoprotective properties of *Dracocephalum rupestre* support its traditional application as a functional beverage with systemic health benefits. Its capacity to modulate oxidative stress and safeguard vital organs underscores its potential for use in preventing and managing oxidative stress-related diseases.

Taken together, these findings substantiate the classification of *Dracocephalum rupestre* as a functional beverage possessing significant antioxidant and health-promoting attributes.

#### 3.3.6. *Dracocephalum komarovi* Lipsky

*Dracocephalum komarovi* has attracted considerable scientific attention due to its abundant phytochemical composition and bioactive compounds with potential pharmacological and antioxidant activities. Research into its phytochemistry has identified various secondary metabolites, including monoterpene glucosides and quinone derivatives, which may contribute to its biological effects [186,187].

Research has documented the isolation of novel monoterpene glucosides demonstrating anti-inflammatory properties, indicating a close correlation between antioxidant and anti-inflammatory mechanisms, as both processes are interconnected via oxidative stress pathways [187]. Furthermore, compounds such as komaroviquinone have been examined for their biological potential, including their participation in redox-related mechanisms and prospective therapeutic applications in diseases associated with oxidative stress [188,189].

Furthermore, trypanocidal constituents isolated from *Dracocephalum komarovi* indicate that this species contains structurally diverse compounds with potential biological activity, including modulation of oxidative stress processes [186].

Although the antioxidant activity of *Dracocephalum komarovi* has not been as extensively studied as in other species, such as *Dracocephalum moldavica* or *Dracocephalum kotschyi*, the presence of chemically diverse secondary metabolites suggests a promising antioxidant potential that warrants further investigation [6,187,189].

#### 3.3.7. *Dracocephalum forrestii* W.W.Sm.

*Dracocephalum forrestii* has been extensively investigated as a prospective source of bioactive compounds exhibiting antioxidant properties, primarily through in vitro cultures and phytochemical analyses. Research indicates that this species can synthesize elevated levels of phenolic compounds, such as rosmarinic acid and salvianolic acid B, recognized for their potent antioxidant effects [101]. Experiments involving transformed shoots and hairy root cultures have demonstrated that environmental factors, particularly light, significantly influence phenolic accumulation and antioxidant activity [190]. These findings imply that controlled cultivation methodologies could enhance the production of antioxidant metabolites.

Quantitative analyses have also confirmed that rosmarinic acid is a major antioxidant, enhancing the species’ ability to scavenge radicals and exert biological effects [101]. In conclusion, *Dracocephalum forrestii* emerges as a promising source of natural antioxidants, especially for biotechnological efforts to boost the production of valuable phenolic compounds.

Further research confirms that extracts from in vitro shoot cultures exhibit potent antioxidant activity, including free radical scavenging and reducing power, which are closely linked to their phenolic content [191]. Multiple assays consistently demonstrate their antioxidant potential, reflecting a strong capacity to neutralize reactive oxygen species.

Research concerning transformed shoots cultivated in bioreactors further corroborates these findings. It demonstrates that the extracts exhibit substantial antioxidant activity, although variations may be observed depending on the culture type and metabolite profile [192]. The presence of both phenolic acids and flavonoid derivatives enhances this activity, suggesting potential synergistic effects among compound classes.

More recent studies confirm that optimized culture conditions, including bioreactors and specific light regimes, can enhance antioxidant potential, with extracts exhibiting strong radical-scavenging activity and inhibiting oxidative processes such as lipid peroxidation [193].

Overall, the data indicate that *Dracocephalum forrestii* is a promising source of natural antioxidants. Its activity is closely associated with phenolic compounds and is affected by cultivation conditions, positioning it as a compelling option for further pharmacological and biotechnological research [191,192,193].

#### 3.3.8. *Dracocephalum foetidum* Bunge

*Dracocephalum foetidum* has garnered scientific interest owing to its diverse phytochemical profile, particularly the presence of monoterpene glycosides, phenylpropanoids, and flavonoid derivatives. These compounds may contribute to its biological activity, including antioxidant effects [68]. Phytochemical investigations have identified several secondary metabolites with potential radical-scavenging capacity, suggesting that this species may help modulate oxidative stress [68].

The antioxidant potential of *Dracocephalum foetidum* is thought to be linked to both direct and indirect mechanisms, such as the neutralization of reactive oxygen species and the augmentation of endogenous antioxidant defense systems [96,99]. Notably, phenylpropanoid derivatives in this species are known for their redox activity and may help stabilize free radicals and prevent oxidative damage [68].

Although the antioxidant activity of *Dracocephalum foetidum* has been less extensively studied than that of other species, such as *Dracocephalum moldavica* or *Dracocephalum kotschyi*, available data indicate that its phytochemical profile supports a potential role as a natural source of antioxidant compounds [68].

Overall, *Dracocephalum foetidum* is a promising yet insufficiently studied species, and additional research is required to comprehensively elucidate its antioxidant mechanisms and potential applications within the pharmaceutical and nutraceutical industries.

#### 3.3.9. *Dracocephalum argunense* Fisch ex. Link and *Dracocephalum integrifolium* Bunge

*Dracocephalum argunense* and *Dracocephalum integrifolium* are less studied species within their genus. However, phytochemical research indicates they contain bioactive compounds with potential antioxidant effects [194]. Reports have identified flavonoids, phenolic compounds, and other secondary metabolites commonly associated with antioxidant activity in plants [2,7]. While comprehensive antioxidant assessments are limited for species such as *Dracocephalum moldavica* or *Dracocephalum kotschyi*, the presence of phenolic compounds suggests they may have free-radical-scavenging capabilities and help modulate oxidative stress [2].

Additionally, their chemical similarity to other members of the Lamiaceae family supports the idea that they could serve as natural antioxidants. Still, further research is necessary to confirm their biological activity and fully characterize their phytochemical profiles [2,7].

In addition to their potential antioxidant activity, *Dracocephalum argunense* has exhibited notable anti-allergic and anti-inflammatory properties in experimental models. Research has indicated that aqueous extracts of this plant inhibit mast-cell-mediated hypersensitivity reactions by reducing histamine release and suppressing systemic and local allergic responses in vivo [195,196]. These effects are linked to the modulation of intracellular calcium levels and the signaling pathways involved in mast cell activation [195].

Furthermore, *Dracocephalum argunense* has been reported to reduce the production of pro-inflammatory cytokines, including tumor necrosis factor-α (TNF-α) and interleukin-6 (IL-6), which play key roles in allergic inflammation [195,196]. The underlying mechanisms include inhibition of transcription factors such as NF-κB and signaling pathways such as p38 MAPK, as well as an increase in intracellular cAMP levels, thereby contributing to the suppression of mast cell degranulation [195,196]. Similarly, *Dracocephalum moldavica* exhibits anti-inflammatory effects by suppressing the TNF-α/NF-κB pathway, thereby contributing to neuroprotection in vascular dementia models, highlighting a shared molecular mechanism with *Dracocephalum argunense* [197].

These findings suggest that, in addition to their potential antioxidant properties, species such as *Dracocephalum argunense* may have therapeutic potential for managing allergic and inflammatory conditions, although further studies are needed to fully elucidate their bioactive compounds and mechanisms of action.

#### 3.3.10. *Dracocephalum officinale* L.

*Dracocephalum officinale* has recently garnered attention as a potential source of valuable antioxidant phytochemicals; however, this species remains significantly less studied than *Dracocephalum moldavica* or *Dracocephalum kotschyi* [198]. A recent phytochemical investigation of the blue-flowered variant cultivated in Ukraine revealed that this species contains both a complex volatile fraction and a substantial array of phenolic acids, underscoring its potential biological significance [198].

Gas chromatography–mass spectrometry analysis revealed 40 volatile compounds in the essential oil, with pinocamphone, isopinocamphone, β-pinene, germacrene D, α-sabinene, myrtenol, and γ-elemene as the major constituents [198]. In parallel, high-performance liquid chromatography analysis of the 80% methanolic extract identified nine phenolic acids, among which rosmarinic acid and caffeic acid were the predominant compounds [198]. Since both rosmarinic acid and caffeic acid are well-recognized antioxidant metabolites, their abundance strongly supports this species’ antioxidant potential [99,102,103,198].

The antioxidant activity of *Dracocephalum officinale* was assessed using the DPPH assay, and the methanolic extract exhibited a moderate free-radical-scavenging effect, with an IC50 of 1.65 mg/mL [198]. Although this activity was labeled as moderate, the findings confirm that the species has a measurable antioxidant capacity, likely linked to its phenolic acid profile, especially its high rosmarinic acid content [198].

Beyond its antioxidant potential, the phytochemical composition of *Dracocephalum officinale* suggests broader pharmacological significance, particularly given the known antimicrobial, anti-inflammatory, and protective effects of several of its volatile and phenolic components [198]. Overall, this species seems to be a promising yet still underexplored member of its genus, and additional research using more antioxidant models, mechanistic assays, and in vivo studies is necessary to better understand its therapeutic potential [198].

#### 3.3.11. *Dracocephalum subcapitatum* Kuntze

*Dracocephalum subcapitatum* is a relatively understudied species within its genus, with limited yet emerging evidence supporting its pharmacological potential, particularly in antioxidant activity and metabolic disorders [199]. Native to northeastern Iran, this plant has been traditionally used, although its therapeutic properties have only recently started to be explored through experimental studies [199].

Phytochemical analysis of hydroalcoholic extracts shows a substantial amount of total phenolic compounds, approximately 70 mg gallic acid equivalents per gram of extract, indicating a strong potential for antioxidant activity [199]. Phenolic compounds are known for their ability to neutralize reactive oxygen species and inhibit lipid peroxidation, which likely explains the biological effects observed in this species [93,96,199].

In vivo experiments using streptozotocin-induced diabetic rat models have shown that *Dracocephalum subcapitatum* extract provides significant antioxidant and protective benefits. Specifically, treatment led to a notable decrease in oxidative stress markers, such as malondialdehyde (MDA), and a significant increase in the plasma total antioxidant capacity [199]. These results suggest that the extract can effectively regulate oxidative stress, a key factor in the development of diabetes and related complications.

Additionally, the extract demonstrated other health benefits, including antihyperglycemic and hypolipidemic effects, demonstrated by reductions in blood glucose, HbA1c, triglycerides, and markers of organ damage [199]. The protective effects on liver, kidney, and pancreatic tissues further support the idea that antioxidant mechanisms are closely tied to its overall therapeutic potential. In summary, although *Dracocephalum subcapitatum* is not yet fully characterized relative to other species in the genus, current evidence suggests it may be a valuable source of natural antioxidants with potential applications in managing metabolic and oxidative stress-related diseases. More phytochemical and clinical research is needed to identify its active constituents and better understand its mechanisms of action [199].

#### 3.3.12. Understudied *Dracocephalum* Species

While many *Dracocephalum* species have been thoroughly studied, information on several taxa remains limited. However, current research shows these plants contain various phytochemicals and have promising antioxidant properties. The species discussed here are grouped together due to the limited evidence available, emphasizing their potential and underscoring the need for further phytochemical and pharmacological research.

*Dracocephalum microphyton* C.Y.Wu & H.W.Li is a less well-known species within the genus, with limited data on its phytochemical makeup and biological effects. This species has mainly been studied from a taxonomic and botanical view, with recent research emphasizing its morphological features, distribution, and evolutionary relationships within the genus *Dracocephalum* [200].

Although detailed phytochemical and pharmacological research is currently limited, its placement within the Lamiaceae family suggests it may contain bioactive compounds typical of related species. These include flavonoids, phenolic acids, and terpenoids, which are well known for their antioxidant activity, particularly in neutralizing reactive oxygen species and protecting cells from oxidative stress.

From a chemotaxonomic perspective, *Dracocephalum microphyton* helps improve understanding of evolutionary relationships within the genus, as variations in morphological and genetic traits may relate to differences in secondary metabolite profiles [200]. Such insights are valuable for guiding future phytochemical studies, especially in identifying species that could be new sources of natural antioxidants.

Given the growing scientific interest in understudied plant species, *Dracocephalum microphyton* is a promising candidate for future research. Comprehensive phytochemical screening and biological testing are essential for assessing its antioxidant potential and potential therapeutic uses. Expanding studies on this species could lead to the discovery of new bioactive compounds and improve understanding of the chemical diversity within the genus *Dracocephalum* [2,200].

Another insufficiently explored species is *Dracocephalum charkeviczii* Prob., a rare medicinal plant native to coastal regions of the Russian Far East, Japan, and China. Available studies have mainly focused on its conservation and in vitro propagation. Nevertheless, phytochemical investigations have identified rosmarinic acid and rabdosiin in its leaves, compounds known for their antioxidant and biological activities. Although current evidence regarding its antioxidant potential remains limited, the presence of these metabolites suggests that *Dracocephalum charkeviczii* represents a promising target for future phytochemical and pharmacological research [201].

Collectively, these findings suggest that antioxidant activity in *Dracocephalum* species is closely linked to phytochemical diversity, thereby supporting further discussion of the mechanisms and biological implications underlying these effects.

#### 3.3.13. Comparative Phytochemical Analysis Among Species

Comparative phytochemical analyses have demonstrated that various *Dracocephalum* species exhibit distinctive chemical profiles, indicative of their ecological adaptations, genetic variability, and evolutionary history [5]. These variations are particularly evident in the distribution and abundance of principal bioactive compounds, including flavonoids, phenolic acids, terpenoids, and other secondary metabolites, which are closely linked to antioxidant activity [5,7].

As summarized in Table 1, different species of the genus are characterized by specific classes of compounds that contribute to their antioxidant potential. For instance, *Dracocephalum moldavica* is particularly rich in flavonoids and phenolic acids, including tilianin and rosmarinic acid, as well as volatile compounds such as monoterpenes and sesquiterpenes, all of which are associated with strong radical scavenging activity and inhibition of oxidative stress [8,21,41,46,57].

Numerous studies have demonstrated that species abundant in flavonoids and phenolic acids, such as *Dracocephalum moldavica* and *Dracocephalum kotschyi*, typically exhibit enhanced antioxidant activity due to the potent radical scavenging capacity of these constituents [21,23,41]. Conversely, species characterized by higher concentrations of volatile terpenoids or other secondary metabolites may exhibit moderate antioxidant effects, often mediated indirectly by modulating oxidative stress pathways [31,68].

These interspecific variations in phytochemical composition may account for the observed differences in antioxidant capacity among *Dracocephalum* species, as evidenced by both in vitro and in vivo studies [21,23,41]. For instance, extracts exhibiting elevated levels of total phenolics and flavonoids invariably show enhanced DPPH and FRAP antioxidant activities, underscoring the pivotal contribution of these compounds to biological efficacy.

In addition to their pharmacological significance, these phytochemical variations are also pertinent to chemotaxonomic investigations, as they offer valuable insights into the relationships among species within the genus [5]. The identification of species-specific chemical markers, such as tilianin in *Dracocephalum moldavica* or xanthomicrol in *Dracocephalum kotschyi*, can facilitate species authentication and quality assurance of plant-derived products [7,13].

Furthermore, integrating phytochemical and biological data facilitates the identification of species with the greatest potential as natural sources of antioxidants. This methodology is especially pertinent for selecting plant material within pharmaceutical, nutraceutical, and food sectors, where both chemical composition and biological activity are critical considerations [7].

In conclusion, comparative phytochemical analysis underscores the importance of understanding species-specific chemical diversity in *Dracocephalum*, which is essential not only for elucidating their biological activity but also for enhancing their use as sources of natural antioxidants.

### 3.4. Mechanisms Underlying Antioxidant Activity

The antioxidant activity of *Dracocephalum* species is primarily attributable to elevated levels of phenolic compounds and flavonoids, which constitute the principal bioactive metabolites identified in numerous species within this genus [2]. These compounds possess multiple hydroxyl groups that can donate hydrogen atoms or electrons, thereby neutralizing reactive oxygen species (ROS) and safeguarding biological systems from oxidative damage [2,202].

A fundamental mechanism underlying their antioxidant efficacy is free radical scavenging, whereby phenolic constituents such as flavonoids and phenolic acids engage directly with ROS, including superoxide, hydroxyl, and peroxyl radicals. This interaction converts these radicals into more stable derivatives and disrupts oxidative chain reactions [2]. Flavonoids, including tilianin, luteolin derivatives, and apigenin derivatives—detected across various *Dracocephalum* species—demonstrate robust free radical scavenging capabilities owing to their polyphenolic framework [7,8]. Another vital mechanism involves metal ion chelation, in which phenolic compounds bind transition metals such as iron and copper, thereby catalyzing the generation of highly reactive hydroxyl radicals via Fenton reactions. By sequestering these metals, they hinder ROS formation and diminish oxidative stress [2], thereby enhancing the antioxidant efficacy of plant extracts.

In addition to direct free-radical scavenging and metal-chelating activities, certain *Dracocephalum* flavonoids may exert antioxidant effects by modulating intracellular signaling pathways. Eriodictyol and eriodictyol-7-O-glucoside isolated from *Dracocephalum rupestre* were reported to activate the Nrf2/ARE pathway, promoting the expression of antioxidant and cytoprotective enzymes, including HO-1, NQO1, and γ-GCS, thereby enhancing cellular resistance to oxidative stress and oxidative damage [182,184].

Furthermore, compounds derived from *Dracocephalum* can inhibit lipid peroxidation, a deleterious process that compromises cellular membranes and dietary lipids. By stabilizing lipid radicals and interrupting oxidative chain reactions, they preserve cellular integrity and enhance biological stability [2]. This attribute is particularly significant in food preservation, where plant-based antioxidants help delay lipid oxidation.

The antioxidant potential of *Dracocephalum* extracts is frequently assessed via in vitro assays such as DPPH radical scavenging, ABTS radical cation bleaching, and FRAP, which rapidly gauge their capacity to neutralize free radicals and reduce oxidized molecules [2,21,41]. For instance, extracts from *Dracocephalum moldavica* seeds, prepared using various methods, exhibit potent antioxidant activity in DPPH and FRAP assays, confirming the presence of active phenolics [21,123,126,203]. These polyphenolic extracts also exhibit notable radical-scavenging and DNA-protective effects, underscoring their antigenotoxic and antioxidant properties [22,41]. In addition, in vivo studies corroborate these findings, demonstrating that consumption of *Dracocephalum moldavica* preparations can bolster antioxidant defenses and influence metabolic pathways associated with oxidative stress [125]. Notably, intake of *Dracocephalum moldavica* tea has been shown to mitigate high-fat diet-induced metabolic disorders and improve lipid metabolism, effects partly attributable to its antioxidant constituents [125].

Recent investigations further reveal that compounds from *Dracocephalum* can modulate cellular signaling pathways implicated in oxidative stress, inflammation, and apoptosis, thereby suggesting protective effects beyond mere free-radical scavenging [8]. In sum, the antioxidant activity of *Dracocephalum* arises from the synergistic actions of various phytochemicals operating through mechanisms such as radical scavenging, metal chelation, inhibition of lipid peroxidation, and regulation of endogenous antioxidant defenses. These mechanisms account for the notable biological activities observed in extracts from multiple *Dracocephalum* species and underscore their potential as natural sources of antioxidants for pharmaceutical, nutraceutical, and food-preservation applications [2].

### 3.5. Towards Q-Marker Identification in Dracocephalum

Although there is growing evidence on the phytochemical composition and biological activities of *Dracocephalum* species, a comprehensive framework that integrates chemical analysis, biological efficacy, and quality control remains insufficiently developed. In recent years, the idea of quality markers (Q-markers) has gained prominence as a key approach for standardizing and evaluating the quality of medicinal plants and phytopharmaceuticals. Q-markers are defined as quantifiable bioactive compounds that are directly linked to the therapeutic effects, safety, and consistency of herbal medicines. Their identification requires integrating phytochemical, pharmacological, and mechanistic data to link specific compounds to biological activity and therapeutic relevance.

The genus *Dracocephalum* is a promising candidate for Q-marker-based standardization because of its significant phytochemical diversity and increasing evidence of its antioxidant-related pharmacological effects. Nonetheless, notable variations in phytochemical composition have been observed across species, geographic locations, growth stages, cultivation methods, and extraction processes, which may affect biological activity and product quality [10,41]. Therefore, identifying reliable Q-markers can support the development of standardized extracts that offer consistent antioxidant and therapeutic benefits.

Among the various phytochemicals found in the genus, phenolic acids and flavonoids stand out as the most promising options for Q-marker selection. Rosmarinic acid is frequently identified as a major phenolic compound in several *Dracocephalum* species, including *Dracocephalum moldavica* and *Dracocephalum forrestii* [42,69,125]. Numerous studies link rosmarinic acid levels with antioxidant activities such as radical scavenging, metal-chelating, and protecting against oxidative DNA damage [42,69]. Additionally, rosmarinic acid is recognized as a biologically significant phenolic metabolite with known antioxidant, anti-inflammatory, and cytoprotective effects, supporting its potential as a primary Q-marker candidate for *Dracocephalum*-based products [69].

Flavonoids represent a second major group of candidate Q-markers. Recent phytochemical studies show that luteolin and apigenin derivatives are among the most common and characteristic flavonoids in the genus [8]. Their widespread presence in various species, along with their known antioxidant and anti-inflammatory effects, makes them valuable for quality evaluation. In *Dracocephalum kotschyi*, luteolin is identified as a major antioxidant component, exhibiting strong free-radical scavenging and significantly contributing to the extract’s antioxidant activity [148]. Similarly, apigenin derivatives have been associated with diverse biological effects and can serve as additional markers that indicate both antioxidant and pharmacological properties [184].

Tilianin (acacetin-7-O-glucoside), one of the most extensively investigated flavonoids in *Dracocephalum*, represents another promising Q-marker candidate. This compound has been reported in significant amounts in *Dracocephalum moldavica* and has attracted considerable attention because of its antioxidant, anti-inflammatory, cardioprotective, neuroprotective, and metabolic regulatory activities [78,101]. Beyond its abundance, tilianin exhibits clear mechanistic links with oxidative stress modulation and cellular protection, supporting its suitability as a bioactivity-oriented quality marker. The growing pharmacological evidence on tilianin further strengthens its potential value for the standardization of *Dracocephalum*-based nutraceuticals and phytopharmaceuticals [78,101].

Additional compounds could also be useful for future Q-marker panels. Phenolic metabolites like caffeic acid, salvianolic acid B, and related phenylpropanoid derivatives have been found in various *Dracocephalum* species and show strong antioxidant properties [125]. Similarly, eriodictyol and eriodictyol-7-O-glucoside extracted from *Dracocephalum rupestre* have exhibited powerful antioxidant effects by activating the Nrf2 signaling pathway, thereby boosting cellular defenses against oxidative stress [182,184]. These results are especially significant because they demonstrate a direct mechanistic link between specific phytochemicals and the body’s natural antioxidant pathways, which is a crucial factor in selecting modern Q-markers.

From a mechanistic perspective, the proposed Q-markers collectively represent the primary antioxidant pathways identified in *Dracocephalum* species. These encompass direct free radical scavenging, metal ion chelation, inhibition of lipid peroxidation, regulation of reactive oxygen species (ROS) production, and activation of endogenous antioxidant defenses such as the Nrf2/ARE pathway [42,148,182,184]. Consequently, the selected compounds not only underscore key chemical constituents but also serve as indicators of biological activity.

Based on the currently available evidence, rosmarinic acid, tilianin, luteolin derivatives, and apigenin derivatives are the most promising Q-marker candidates in *Dracocephalum* species. Nevertheless, future studies integrating metabolomics, bioactivity-guided fractionation, pharmacokinetic studies, and comparative analyses across multiple species are necessary to confirm their validity and develop standardized quality control methods. An integrated Q-marker system such as this would support the creation of reliable, effective, and scientifically validated *Dracocephalum* products for pharmaceutical, nutraceutical, and functional food uses.

### 3.6. Comparative Analysis of Antioxidant Activity Among Dracocephalum Species

Considering the antioxidant mechanisms discussed above, a comparative evaluation among *Dracocephalum* species could provide further insight into variations in their phytochemical profiles, antioxidant capacity, and biological significance. A comprehensive analysis of the available literature indicates that antioxidant activity is a common and defining feature across numerous *Dracocephalum* species; however, its intensity, underlying mechanisms, and biological relevance vary considerably depending on species-specific phytochemical composition (Table 2). These variations are primarily attributed to differences in the content and diversity of phenolic compounds, flavonoids, and other secondary metabolites [2,7].

Among the investigated species, *Dracocephalum moldavica* and *Dracocephalum kotschyi* are the most extensively studied and consistently demonstrate strong antioxidant activity. This effect is largely associated with their high concentrations of flavonoids and phenolic acids, particularly rosmarinic acid, tilianin, and methoxylated flavones such as xanthomicrol [2,13,21,41]. These compounds exhibit potent radical scavenging activity, metal-chelating properties, and the ability to inhibit lipid peroxidation, thereby contributing to significant antioxidant effects in both in vitro and in vivo models [21,41].

In contrast, species such as *Dracocephalum heterophyllum* and *Dracocephalum tanguticum* have been less extensively investigated, yet they display promising antioxidant-related biological activities. In these species, antioxidant effects are associated with specific secondary metabolites, including phenolic alkaloids and flavonoid glycosides, which have been shown to neutralize reactive oxygen species and protect cellular structures against oxidative damage [18,19,26].

*Dracocephalum rupestre*, traditionally consumed as Maojian herbal tea, represents an important example of a functional plant with antioxidant potential. Its rich content of phenolic compounds and flavonoids contributes to its ability to scavenge free radicals and support health-promoting effects, highlighting its relevance in nutraceutical and dietary applications [20,28]. The presence of bioactive flavonoids, such as eriodictyol, further supports its role in modulating oxidative stress and exerting neuroprotective effects [204].

Other species, including *Dracocephalum komarovi* and *Dracocephalum forrestii*, demonstrate antioxidant potential that is closely linked to their unique phytochemical profiles. In *Dracocephalum komarovi*, monoterpene glucosides and quinone derivatives are believed to contribute to redox-related mechanisms and indirect antioxidant effects [186,187,188,189]. Meanwhile, *Dracocephalum forrestii* is characterized by high levels of phenolic acids, such as rosmarinic acid and salvianolic acid B, whose accumulation can be enhanced through controlled in vitro culture systems [101,190].

Besides these well-studied species, several lesser-known members of the genus have recently gained interest. *Dracocephalum officinale* shows moderate antioxidant activity, as evidenced by DPPH radical-scavenging assays, which correlate with its phenolic acid content, particularly rosmarinic and caffeic acids [198]. Although its antioxidant effect is less potent than that of highly active species, its phytochemical profile indicates potential for further exploration in pharmacological development. Similarly, *Dracocephalum subcapitatum* has shown notable antioxidant-related biological effects in experimental models. Its relatively high phenolic content helps reduce oxidative stress markers and boost total antioxidant capacity in vivo, especially under conditions such as diabetes [199]. These findings emphasize its potential role in managing disorders linked to oxidative stress.

In contrast, species such as *Dracocephalum microphyton* remain largely unexplored in terms of phytochemistry and antioxidant testing. Current knowledge is largely limited to taxonomic and phylogenetic studies; however, based on its classification within the Lamiaceae family, it likely contains bioactive compounds with potential antioxidant properties [2,200]. This highlights the need for future research focusing on underexplored species within the genus.

An important factor influencing antioxidant activity across *Dracocephalum* species is the extraction method and processing technique employed. Studies have demonstrated that solvent polarity, extraction conditions, and methodology significantly affect the yield of phenolic compounds and, consequently, the measured antioxidant capacity [21,96]. For example, polar solvents typically result in higher phenolic content and stronger radical scavenging activity compared to non-polar solvents [21].

Additionally, environmental factors, cultivation practices, and developmental stages play a crucial role in regulating the biosynthesis of antioxidant metabolites. External stimuli such as light, nanoparticles, and elicitors have been shown to enhance the accumulation of phenolic compounds and improve antioxidant responses in several species [23,33,190,205].

Overall, comparative analysis highlights that the antioxidant potential of *Dracocephalum* species is strongly dependent on their phytochemical diversity and environmental interactions. While certain species, such as *Dracocephalum moldavica* and *Dracocephalum kotschyi*, stand out as particularly rich sources of natural antioxidants, other species remain underexplored and may be valuable candidates for future pharmacological and nutraceutical applications. Further research is required to elucidate the specific mechanisms of action of individual compounds and to validate their therapeutic potential in clinical settings [2].

### 3.7. Innovative and Emerging Research Approaches in Dracocephalum

Recent advancements in phytochemical, pharmacological, and biotechnological research have catalyzed the development of innovative techniques to enhance the extraction, characterization, and application of bioactive compounds from *Dracocephalum* species. These methodologies are particularly significant for augmenting the antioxidant capacity of plant-based compounds and expanding their application across pharmaceutical, nutraceutical, and industrial fields [2].

#### 3.7.1. Advanced Analytical and Metabolomic Approaches

Modern analytical techniques have greatly enhanced our understanding of the chemical complexity of *Dracocephalum* species. Advanced methods such as UPLC, LC–MS, and metabolomic profiling enable thorough identification of phenolic compounds, flavonoids, and other secondary metabolites that contribute to antioxidant activity [7,20].

These techniques enable detailed profiling of phytochemicals and help link chemical composition with biological effects. Specifically, metabolomic analyses provide a comprehensive view of plant metabolism, aiding the discovery of biomarkers associated with antioxidant and other pharmacological properties [20].

#### 3.7.2. In Silico and Molecular Modeling Approaches

Beyond experimental research, computational methods such as molecular docking, density functional theory (DFT), and network pharmacology are increasingly utilized to investigate the mechanisms through which *Dracocephalum* compounds exert their effects [206,207]. These in silico approaches facilitate the prediction of interactions between phytochemicals and biological targets implicated in oxidative stress and disease.

For example, flavonoids and phenolic compounds have been shown to bind to enzymes and receptors implicated in oxidative damage, thereby elucidating their antioxidant and therapeutic properties [206,207]. Furthermore, computational modeling can accelerate drug discovery by identifying promising bioactive compounds and supporting subsequent experimental validation.

#### 3.7.3. Nanotechnology and Delivery Systems

**Figure 14:**
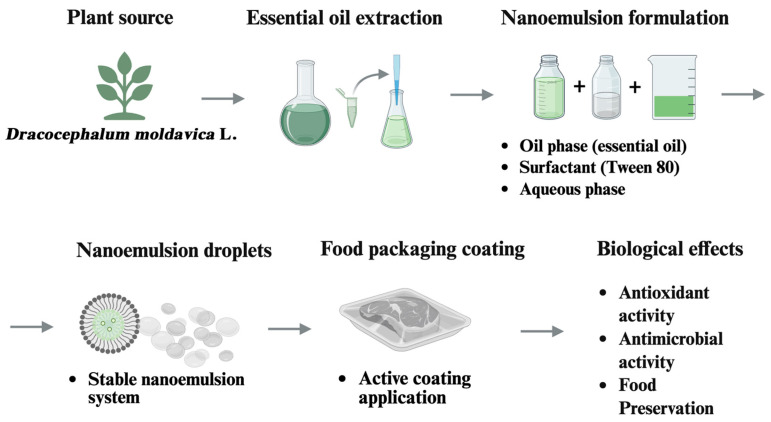
Preparation and application of *Dracocephalum moldavica* essential oil nanoemulsion utilized in active food packaging systems. The nanoemulsion formulation enhances the stability, bioavailability, antioxidant activity, and antimicrobial properties of the essential oil, thereby improving food preservation [14].

Nanotechnology-based approaches are promising for enhancing the stability, bioavailability, and biological activity of compounds derived from *Dracocephalum*. Notably, nanoemulsions and nanoparticle delivery systems are particularly relevant. For instance, nanoemulsion-based formulations, consisting of essential oil dispersed in aqueous systems using surfactants, have demonstrated improved antioxidant and antimicrobial performance, contributing to enhanced oxidative stability and prolonged shelf life of food products (Figure 14) [14]. These findings further emphasize the multifunctional relevance of *Dracocephalum moldavica* essential oil as a natural source of antioxidant compounds with potential applications in food technology and preservation strategies [14].

#### 3.7.4. Nanoparticles and Elicitor-Based Enhancement of Phytochemicals

Recent research has demonstrated that the use of nanoparticles and elicitors can substantially increase secondary metabolite production in *Dracocephalum* species. Treatments with compounds such as zinc oxide or copper oxide nanoparticles have been shown to stimulate the accumulation of phenolic compounds and enhance antioxidant enzyme activity [33,132,205].

Similarly, elicitors such as chitosan nanoparticles have been employed in plant cell cultures to provoke stress responses that enhance the synthesis of bioactive compounds, including flavonoids and phenolic acids [205,208]. These strategies offer a promising approach for augmenting the yield and quality of antioxidant compounds in plant systems.

Additionally, plant extracts from *Dracocephalum moldavica* have been successfully used in the green synthesis of metallic nanoparticles, especially silver nanoparticles, serving as both reducing and stabilizing agents. This environmentally friendly method enables the rapid production of nanoparticles with controlled size and shape while avoiding the use of toxic chemicals [209]. Furthermore, these biosynthesized nanoparticles demonstrate notable biological activities, including antibacterial effects, further emphasizing the multifunctional potential of *Dracocephalum*-derived compounds in nanobiotechnology applications [205,209]. 

These strategies offer a promising approach to increasing the yield and quality of antioxidant compounds in plant systems.

#### 3.7.5. Biotechnological Approaches and Sustainable Production

Biotechnological methods such as plant tissue culture, callus cultures, and cell suspension cultures have become increasingly popular as sustainable approaches for producing valuable secondary metabolites from *Dracocephalum* species [210]. These techniques allow for the controlled production of bioactive compounds regardless of environmental conditions, reducing dependence on natural plant populations. In vitro systems also enable precise control of growth parameters and elicitation strategies to boost yields of antioxidant compounds, while genetic and metabolic engineering approaches can further enhance the biosynthesis of targeted phytochemicals. Additionally, accurate molecular authentication methods are vital for correctly identifying *Dracocephalum* species used in biotechnological processes, preventing adulteration and ensuring consistent phytochemical production [211].

#### 3.7.6. Future Perspectives: AI, Multi-Omics, and Translational Research

Spray-drying methods have been increasingly optimized to enhance the stability and usability of *Dracocephalum moldavica* extracts. This process turns liquid extracts into stable powders, preserving key phytochemicals, such as phenolic compounds and flavonoids, that contribute to antioxidant activity [212]. Studies on process optimization show that factors such as inlet temperature, airflow rate, and carrier concentration significantly impact the physicochemical properties and antioxidant capacity of the final product. These findings highlight the importance of precise control over processing conditions to protect bioactive compounds and enable large-scale industrial use [213].

The incorporation of advanced analytical techniques, computational modeling, nanotechnology, and biotechnology exemplifies a multidisciplinary approach to *Dracocephalum* research. In this context, standardized extracts such as Rosmatin, characterized by a high content of rosmarinic acid and flavonoids, have demonstrated notable anti-inflammatory and gastroprotective effects, supporting their potential therapeutic applications [212]. Ongoing research in this domain is anticipated to facilitate the discovery of new compounds, optimize delivery systems, and advance applications within pharmaceutical, nutraceutical, and industrial sectors [2]. These advances highlight the growing importance of bioactive compounds from *Dracocephalum* in translational and industrial contexts, while also pointing to the necessity for more comprehensive and predictive research approaches that enhance phytochemical analysis, biological understanding, and application-focused development.

Recent advancements in artificial intelligence (AI), machine learning, and multi-omics technologies offer promising opportunities to enhance the understanding, prediction, and practical application of bioactive compounds in *Dracocephalum* species. Machine learning methodologies have demonstrated considerable potential for forecasting key secondary metabolites in *Dracocephalum moldavica*, including compounds associated with stress tolerance and phytochemical responses, while also reducing reliance on labor-intensive, time-consuming, and chemically demanding analytical techniques [214]. Predictive models, particularly those based on neural networks, can support the estimation of vital biochemical traits, improve the efficiency of phytochemical screening, and foster the sustainable cultivation of medicinal plants through more rapid and precise data-driven assessments [214].

In parallel, recent advancements in genomics, transcriptomics, metabolomics, and integrated multi-omics methodologies have the potential to substantially augment our comprehension of phytochemical diversity, antioxidant compounds, and biosynthesis pathways in *Dracocephalum* species [215]. The integration of multi-omics approaches has previously contributed to the identification of genes involved in flavonoid glycosylation and tilianin biosynthesis in *Dracocephalum moldavica*, thereby enhancing the mechanistic understanding of biologically active metabolites and their biosynthetic regulation [215]. These methodologies may facilitate more precise identification of antioxidant phytochemicals, accelerate the discovery of therapeutically significant metabolites, and bolster translational initiatives in pharmaceuticals, nutraceuticals, and functional foods. Overall, the combination of artificial intelligence prediction, machine learning, and multi-omics strategies could serve as a pivotal pathway for the advancement of *Dracocephalum* research and the enrichment of scientific knowledge regarding its antioxidant properties.

### 3.8. Industrial and Nutraceutical Applications

The innovative and emerging research approaches discussed above further support the practical translation of bioactive compounds derived from *Dracocephalum* into industrial, nutraceutical, and pharmaceutical applications. Species of the genus *Dracocephalum* have attracted increasing scholarly interest owing to their extensive potential applications in the industrial, pharmaceutical, and nutraceutical sectors. This heightened interest is primarily due to their rich phytochemical profile, which includes flavonoids, phenolic acids, terpenoids, and essential oil constituents [216]. These compounds are linked to various biological activities, including antioxidant, antimicrobial, anti-inflammatory, and cardioprotective effects [2,7,217].

The diversity of these bioactive compounds positions *Dracocephalum* species as valuable natural resources for developing functional products, ranging from food additives and nutraceuticals to pharmaceutical formulations and cosmetic ingredients [2].

#### 3.8.1. Functional Foods and Herbal Teas

Various *Dracocephalum* species are traditionally used to prepare herbal infusions and functional beverages, particularly in regions with a longstanding history of medicinal plant use [3]. These products are increasingly recognized not only for their sensory attributes but also for their potential health benefits.

A noteworthy example is Maojian herbal tea, derived from *Dracocephalum rupestre*, which is extensively consumed in China and esteemed for its distinctive aroma and bioactive composition [20,28]. Metabolomic investigations have demonstrated that this tea encompasses a broad spectrum of phenolic compounds and flavonoids, which substantially contribute to its antioxidant properties and potential health advantages [20].

Additionally, the chemical composition and biological effects of Maojian tea can vary with processing techniques, such as those used in green and black tea manufacturing, which affect the levels of active compounds [20]. These results emphasize how processing conditions play a crucial role in shaping the functional qualities of plant-based drinks.

Another significant species is *Dracocephalum moldavica*, whose aerial parts are frequently used to prepare aromatic herbal infusions and are reported to possess digestive, anxiolytic, and antioxidant properties [3,218]. Experimental investigations have also demonstrated that intake of *Dracocephalum moldavica* tea may improve metabolic parameters and reduce oxidative stress in high-fat diet models, thereby reinforcing its potential as a functional beverage [125].

#### 3.8.2. Nutraceuticals and Dietary Supplements

The rising demand for natural health-enhancing products has prompted further investigation into *Dracocephalum* species as sources of nutraceutical ingredients and dietary supplements [2]. The presence of bioactive compounds, such as tilianin, rosmarinic acid, and other flavonoids, has been linked to numerous health benefits, including antioxidant, cardioprotective, and anti-inflammatory effects [7,8].

Tilianin, in particular, has been extensively investigated for its role in cardiovascular protection, including its ability to modulate inflammatory pathways and oxidative stress mechanisms [8,219]. Similarly, rosmarinic acid is recognized for its strong radical-scavenging activity and capacity to protect biological systems from oxidative damage [7].

These compounds may be integrated into nutraceutical formulations intended to mitigate oxidative stress, promote cardiovascular health, and enhance overall physiological functions. Additionally, the synergistic effects of various phytochemicals present in *Dracocephalum* extracts may enhance their effectiveness compared with isolated compounds [2,220].

#### 3.8.3. Essential Oil Industry

Essential oils derived from various *Dracocephalum* species have garnered significant interest owing to their aromatic properties and potential industrial applications [17,30]. These oils are abundant in monoterpenes, sesquiterpenes, and oxygenated derivatives, which contribute to their distinctive fragrance and biological activities [30,31].

Beyond their applications in perfumery and cosmetics, these essential oils possess antimicrobial and antioxidant properties, making them suitable as natural preservatives in food systems [2,221]. For example, essential oil derived from *Dracocephalum moldavica* has been integrated into nanoemulsions and active packaging systems, thereby enhancing food preservation and extending shelf life [14].

Furthermore, research indicates that essential oils derived from *Dracocephalum* species may exert protective effects against oxidative stress in biological systems, thereby supporting their use in functional products and pharmaceutical formulations [31].

#### 3.8.4. Cosmetic and Pharmaceutical Applications

The extensive phytochemical profile of *Dracocephalum* species underscores their potential for integration into cosmetic and pharmaceutical products [2]. The presence of antioxidant and anti-inflammatory compounds makes these plants particularly suitable for formulations designed to safeguard the skin against oxidative stress and environmental damage (exposome).

Phenolic compounds and flavonoids derived from these species may help prevent premature skin aging by neutralizing reactive oxygen species and mitigating inflammation [7]. These attributes underpin their application in anti-aging, protective, and therapeutic cosmetic formulations.

Recent advancements in nanotechnology have created new opportunities to utilize compounds derived from *Dracocephalum*. For example, carbon dots synthesized from *Dracocephalum moldavica* have demonstrated promising therapeutic properties, particularly in modulating inflammation and oxidative stress in biomedical applications [15]. Additionally, biotechnological methods such as plant tissue culture and elicitor-based systems may promote the sustainable production of bioactive compounds, thereby further increasing the industrial potential of *Dracocephalum* species [132,210].

Additional experimental and clinical findings support the dermatological potential of *Dracocephalum moldavica*. Its extracts have been shown to upregulate key collagen-related genes (*COL3A1*, *COL6A1*, *COL16A1*) and promote extracellular matrix organization, which are essential for maintaining skin integrity and elasticity [222]. Oral supplementation has also been reported to significantly improve skin hydration and increase dermal thickness, reinforcing its value as a natural anti-aging and collagen-enhancing agent [223].

### 3.9. Research Gaps

Despite the promising biological activities and the increasing industrial applications previously referenced, substantial research gaps and methodological limitations remain. These challenges impede a more comprehensive understanding and restrict the broader utilization of *Dracocephalum* species. Presently, only a limited number of species, such as *Dracocephalum moldavica* and *Dracocephalum kotschyi*, have been extensively examined, leaving many other species insufficiently explored or entirely uncharacterized in terms of their phytochemical composition and biological effects [2]. Future investigations should therefore prioritize less-explored *Dracocephalum* species to obtain a more comprehensive view of the genus diversity and bioactive potential.

Another significant limitation is the insufficiency of clinical evidence supporting the therapeutic applications of *Dracocephalum* species. Although numerous in vitro and in vivo studies have demonstrated promising antioxidant, anti-inflammatory, and pharmacological effects, well-designed clinical trials remain limited [2]. Such studies are vital to substantiate the efficacy, safety, and potential health benefits of these plant-derived compounds in human subjects.

Furthermore, the standardization of plant extracts poses a substantial challenge to their development into pharmaceutical and nutraceutical products. Variability in phytochemical composition, influenced by environmental conditions, geographical origin, cultivation practices, and extraction methods, can significantly affect biological activity, particularly antioxidant capacity [7]. Consequently, it is imperative to establish standardized protocols for extraction, characterization, and quality control to guarantee reproducibility and consistency of results.

Toxicological evaluation remains an area requiring additional scrutiny. Although *Dracocephalum* species are traditionally regarded as safe, comprehensive research concerning their toxicity, potential adverse effects, and long-term safety remains limited, particularly for concentrated extracts and isolated compounds intended for pharmaceutical use [2].

Future research should adopt an integrated, multidisciplinary approach that merges phytochemical, pharmacological, and clinical studies. Furthermore, employing advanced technologies such as metabolomics, nanotechnology, and biotechnology can significantly improve the discovery, production, and delivery of bioactive compounds. Developing sustainable cultivation methods and biotechnological systems, such as plant tissue cultures, will be essential to ensure a consistent supply of high-quality plant material [209].

Overall, addressing these research gaps will be essential to fully harness the potential of *Dracocephalum* species as valuable sources of natural antioxidants and to advance their applications in medicine, nutrition, and industry.

#### Clinical Research Gaps

Although most evidence on *Dracocephalum* species comes from phytochemical investigations and preclinical research, a few human clinical trials have provided initial support for their therapeutic potential. However, the current clinical evidence is limited and requires validation through larger, more rigorous studies.

A randomized, double-blind, placebo-controlled clinical trial conducted in Iran evaluated the effectiveness of *Dracocephalum kotschyi* extract in patients with IBS-D [153]. The study involved 76 adults aged 18–50 years who met the Rome III diagnostic criteria for irritable bowel syndrome. Participants took 75 mg capsules of *Dracocephalum kotschyi* dry extract or placebo three times daily for four weeks and were followed for another four weeks. Outcomes were measured using the IBS Severity Index and the IBS-QOL questionnaire. The treatment group showed significant improvements in symptoms, including reduced abdominal discomfort, fewer bowel disturbances, lower disease severity, and better quality-of-life scores. Some benefits decreased after stopping treatment, but overall, the effects remained better than placebo. The authors suggested that *Dracocephalum kotschyi* extract might be a promising additional therapy for IBS-D, owing to its anti-inflammatory, antispasmodic, antioxidant, and gastroprotective effects [153].

Beyond gastrointestinal applications, a double-blind, randomized, placebo-controlled clinical trial evaluated the anti-aging and skin health effects of *Dracocephalum moldavica* extract in 103 healthy women aged 35–65 years [222]. Participants took either 100 mg of the extract or a placebo daily for 12 weeks. Results showed notable increases in skin hydration at 4, 8, and 12 weeks. Ultrasound scans indicated improvements in dermal and overall skin thickness, pointing to better skin structural integrity. In vitro tests with human dermal fibroblasts revealed increased expression of genes related to extracellular matrix and collagen production, including COL3A1, COL6A1, COL16A1, and BGN. The extract, rich in flavonoid-glucuronides with antioxidant and anti-inflammatory properties, likely contributed to these benefits [222]. The study suggests that *Dracocephalum moldavica* is a promising plant-based nutricosmetic ingredient that can boost collagen synthesis, enhance skin hydration, and support healthy skin aging.

Additionally, a randomized, double-blind, placebo-controlled trial investigated the effects of *Dracocephalum* extract on sleep quality in postmenopausal women aged 50–60 years [224]. The authors did not clearly identify the *Dracocephalum* species used or offer detailed phytochemical information about the extract, potentially impacting reproducibility and comparability with other clinical research. Participants in the intervention group took 250 mg capsules of the extract twice daily for a month, while the control group received starch capsules as a placebo. Sleep quality was measured using the Pittsburgh Sleep Quality Index (PSQI). Results showed a significant reduction in the mean PSQI score in the treatment group, from 12.69 ± 3.98 at baseline to 8.58 ± 1.97 by the end, with only slight changes in the placebo group. The difference between the groups after treatment was statistically significant. The authors proposed that the plant’s effects might stem from its impact on neurochemical pathways involved in sleep regulation, including modulation of γ-aminobutyric acid (GABA) metabolism and neurotransmitter signaling [224]. These findings suggest that *Dracocephalum* extract could be a safe and effective herbal option for enhancing sleep quality and reducing sleep issues in postmenopausal women.

Despite these promising findings, the clinical evidence for the medicinal use of *Dracocephalum* species is still limited in both scope and quality. Additionally, human studies have mainly concentrated on *Dracocephalum kotschyi* and *Dracocephalum moldavica*, leaving many other species unexamined. Critical factors such as long-term safety, pharmacokinetics, bioavailability of active compounds, optimal dosing strategies, herb–drug interactions, and efficacy across different populations remain poorly understood. Therefore, larger multicenter randomized controlled trials with extended follow-up periods are needed to confirm the therapeutic benefits observed to date and to support the development of evidence-based pharmaceuticals, nutraceuticals, and functional foods derived from *Dracocephalum* species.

### 3.10. Future Directions

While considerable progress has been made in characterizing the phytochemicals, assessing biological effects, and exploring the pharmacology of *Dracocephalum* species, several crucial research challenges persist. Recent developments in genomics, metabolomics, biotechnology, and pharmaceutical sciences provide new opportunities to better understand the molecular mechanisms underlying the biological activities of *Dracocephalum* compounds. Future research should go beyond traditional phytochemical screening and adopt integrated approaches that connect genetic regulation, metabolite production, bioavailability, and clinical outcomes. In particular, employing advanced omics technologies, exploring plant–microbiota interactions, creating innovative delivery systems, and conducting well-structured clinical trials are expected to greatly improve the translational potential of *Dracocephalum* studies, helping to develop evidence-based therapeutic and nutraceutical products [225,226,227].

#### 3.10.1. Multi-Omics and Single-Cell Sequencing Approaches

Recent genomic and transcriptomic research has greatly enhanced our understanding of the biosynthetic pathways that produce biologically active compounds in *Dracocephalum* species. Transcriptome analysis of *Dracocephalum tanguticum* has identified key genes involved in rosmarinic acid synthesis, offering valuable insights into the regulation of phenolic compound production [226]. Likewise, transcriptomic studies of *Dracocephalum kotschyi* have helped identify candidate genes linked to the biosynthesis of methoxylated flavonoids, including those with known antioxidant and anticancer effects [227]. More recently, the creation of chromosome-level and telomere-to-telomere genome assemblies for *Dracocephalum moldavica* and *Dracocephalum rupestre* has provided essential genetic resources for exploring secondary metabolism, functional genomics, and molecular breeding approaches [225,228].

Future research should focus on combining genomics, transcriptomics, metabolomics, and proteomics to create comprehensive multi-omics platforms for *Dracocephalum* species. These approaches could reveal key regulatory networks that govern the biosynthesis of flavonoids, phenolic acids, terpenoids, and other important metabolites. Moreover, advanced techniques such as single-cell RNA sequencing and spatial transcriptomics might identify cell-specific gene expression patterns and metabolite distributions, providing new insights into the spatial organization of secondary metabolite production. While these technologies have not yet been applied to *Dracocephalum* species, their use could accelerate the discovery of new bioactive compounds and enhance metabolic engineering efforts to increase the production of valuable phytochemicals [225,226,227,228].

#### 3.10.2. Gut Microbiota Interactions and Functional Foods

Recent research suggests that the biological impact of plant-derived polyphenols is predominantly influenced by their interactions with gut microbiota. Flavonoids frequently undergo significant microbial biotransformation, yielding metabolites with varying bioavailability and biological activity. Additionally, these compounds can modify the composition and functions of intestinal microbial communities, supporting gastrointestinal health and overall wellness [229,230]. Since *Dracocephalum* species are rich in flavonoids, rosmarinic acid, and other phenolic compounds, investigating their interactions with the gut microbiome presents a valuable research opportunity.

Particular attention should be paid to rosmarinic acid, a key phenolic component found in several *Dracocephalum* species. Recent studies indicate that rosmarinic acid can decrease intestinal inflammation, enhance barrier integrity, and influence microbial populations by specifically targeting gut microbiota [231]. Additionally, emerging evidence suggests that flavonoids impact not only gut health but also the gut–brain axis, influencing cognitive functions, neuroinflammation, and neurological disorders via microbiota-mediated pathways [230]. Future studies should explore whether phytochemicals derived from *Dracocephalum* may function as ingredients in functional foods to modify gut microbiota composition and improve overall health. Human intervention trials evaluating microbial metabolites, diversity, and health outcomes would be particularly advantageous in this context [229,231].

#### 3.10.3. Nanotechnology and Advanced Delivery Systems

Despite the impressive pharmacological potential of flavonoids and phenolic compounds from *Dracocephalum*, their practical use is often hindered by poor water solubility, low stability, fast metabolism, and limited bioavailability. These issues can significantly diminish the therapeutic effects of otherwise promising bioactive ingredients. Recently, nanotechnology-based delivery systems have become effective solutions for addressing these problems by increasing stability, improving intestinal absorption, extending circulation time, and providing controlled release of active substances [232,233]. Therefore, incorporating nanotechnology into research on *Dracocephalum* represents a key step toward developing more effective pharmaceutical and nutraceutical products.

Encouraging evidence has been documented for *Dracocephalum moldavica*, in which solid lipid nanoparticles loaded with total flavonoid extracts exhibited enhanced physicochemical properties and markedly augmented cardioprotective activity compared with conventional extracts in experimental models of myocardial ischemia–reperfusion injury [232]. Furthermore, recent investigations suggest that nanoparticle-based approaches can stimulate plant growth, increase flavonoid accumulation, enhance antioxidant capacity, and improve essential oil production in *Dracocephalum kotschyi*. These findings highlight the potential of nanotechnology not only for drug delivery but also for the cultivation of medicinal plants and the biosynthesis of metabolites [234]. Future research should focus on nanoemulsions, lipid nanoparticles, polymeric nanocarriers, phytosomes, and other sophisticated delivery systems specifically tailored for *Dracocephalum*-derived compounds. Such approaches may enhance bioavailability, facilitate targeted delivery, reduce dosages, and augment therapeutic efficacy, thereby advancing next-generation phytopharmaceuticals [232,233,234].

#### 3.10.4. Clinical Validation and Translational Research

Although numerous in vitro and in vivo studies have demonstrated antioxidant, anti-inflammatory, antimicrobial, cardioprotective, neuroprotective, and anticancer effects of *Dracocephalum* species, the clinical application of these findings has been limited. A key obstacle is the lack of standardized herbal preparations, which hampers comparison of results across studies and reduces reproducibility. Recent studies emphasize the need for developing validated standardization protocols, quality-control measures, and consistent dosage forms for *Dracocephalum*-based products [235,236]. These initiatives are crucial to ensure uniform phytochemical content, biological effectiveness, and safety.

Future translational research should focus on identifying reliable phytochemical markers, developing standardized extraction techniques, and comprehensively characterizing bioactive compounds. It is equally imperative to pursue pharmacokinetic, toxicological, and dose–response investigations to enhance understanding of the absorption, metabolism, and long-term safety of *Dracocephalum* preparations. Furthermore, forthcoming clinical studies must incorporate precise botanical identification of the species, coupled with detailed phytochemical profiling and standardization of the extracts employed. These measures are vital for improving reproducibility and facilitating meaningful comparisons across clinical research [235,236].

Another key research focus is the conservation and utilization of genetic diversity within the *Dracocephalum* genus. There is notable variation among populations in morphology, essential oil composition, and phytochemical profiles, indicating a strong potential to select elite chemotypes with enhanced medicinal properties [16]. The integration of genomic data, phytochemical analysis, breeding initiatives, and clinical studies could facilitate the development of standardized, evidence-based pharmaceuticals, nutraceuticals, and functional foods derived from *Dracocephalum* species. Large-scale, multicenter randomized controlled trials, supported by comprehensive phytochemical standardization and biomarker-driven methodologies, will be imperative for confirming the genus’ therapeutic capabilities and for transitioning its applications from traditional practices to contemporary, evidence-based products [16,235,236].

## 4. Conclusions

Taken together, the available evidence discussed throughout this review highlights both the scientific relevance and practical potential of *Dracocephalum* species as important sources of antioxidant phytochemicals. The genus *Dracocephalum* constitutes a highly valuable and chemically diverse group of medicinal plants with considerable potential as sources of natural antioxidants. The comprehensive body of research reviewed herein underscores the richness of the phytochemical composition, encompassing flavonoids, phenolic acids, terpenoids, lignans, and other secondary metabolites. Notably, flavonoids and phenolic acids—especially tilianin, luteolin, and apigenin derivatives, as well as rosmarinic acid—are identified as primary contributors to the antioxidant capacity observed across various species [7,8].

The antioxidant activity of *Dracocephalum* species is extensively supported by experimental evidence demonstrating their capacity to neutralize free radicals, regulate oxidative stress pathways, and safeguard biological systems from oxidative damage. These effects are closely linked to the total phenolic and flavonoid content of plant extracts, thereby underscoring the pivotal role of these compounds in determining biological efficacy. Additionally, the variety of mechanisms involved—including radical scavenging, metal-ion chelation, and modulation of oxidative enzymes—indicates that compounds derived from *Dracocephalum* may act through multiple complementary pathways.

Comparative analyses indicate that the antioxidant potential varies substantially among different species, reflecting disparities in phytochemical composition and ecological adaptation. While well-documented species such as *Dracocephalum moldavica* and *Dracocephalum kotschyi* demonstrate consistently high antioxidant activity, other species—including *Dracocephalum heterophyllum*, *Dracocephalum tanguticum*, *Dracocephalum foetidum*, and *Dracocephalum komarovi*—also display promising biological properties that have not been sufficiently investigated. This underscores the need to broaden research beyond the most extensively studied species.

In addition to their pharmacological significance, *Dracocephalum* species exhibit substantial potential for practical applications across multiple sectors. Their incorporation into functional foods, herbal teas, dietary supplements, and natural preservatives underscores their versatility and increasing significance within the nutraceutical and food industries. Furthermore, the use of essential oils and bioactive extracts in cosmetic formulations and pharmaceutical products underscores their industrial relevance.

Recent advances in analytical techniques, metabolomics, nanotechnology, and biotechnology have substantially improved the understanding and application of *Dracocephalum* species. These pioneering methodologies have facilitated more accurate identification of bioactive compounds, optimized extraction processes, and the development of sophisticated delivery systems that enhance bioavailability and stability. Such advancements are crucial for translating laboratory research into practical, real-world applications.

Nevertheless, despite these advancements, numerous significant challenges persist. The paucity of clinical studies hampers the comprehensive validation of the therapeutic efficacy of these plants in humans. Furthermore, variability in phytochemical composition, influenced by environmental conditions and extraction techniques, complicates standardization and reproducibility. Resolving these issues will be essential for the development of safe, efficacious, and standardized products derived from *Dracocephalum* species.

From a future perspective, research should concentrate on elucidating the molecular mechanisms underlying the biological activities of individual compounds, investigating synergistic interactions within complex plant extracts, and expanding studies to underexplored species. Additionally, sustainable cultivation practices and biotechnological production systems are imperative to guarantee the consistent availability of high-quality plant material.

In conclusion, the genus *Dracocephalum* represents a substantial and promising source of natural antioxidants, possessing considerable potential for future pharmaceutical, nutraceutical, and industrial applications. Ongoing interdisciplinary research encompassing phytochemistry, pharmacology, biotechnology, and clinical science will be essential to unlocking the full therapeutic and commercial potential of these plants, thereby supporting their sustainable and effective utilization.

## Figures and Tables

**Table 1:** Major Phytochemicals Identified in *Dracocephalum* Species and Their Relevance to Antioxidant Activity.

Species	Major Compounds	Compound Class	Contribution to Antioxidant Activity	References
*Dracocephalum moldavica* L.	Tilianin, rosmarinic acid, luteolin derivatives, monoterpenes, sesquiterpenes	Flavonoids, phenolic acids, volatile compounds	Strong radical scavenging activity; inhibition of oxidative stress and lipid peroxidation	[7,8,21,41,45,46,51,57]
*Dracocephalum kotschyi* Boiss.	Xanthomicrol, luteolin, apigenin derivatives	Methoxylated flavones	Antioxidant activity associated with flavone structure and ROS scavenging	[13,23,25]
*Dracocephalum heterophyllum* Benth.	Antioxidative flavonoids, phenolic alkaloids	Flavonoids, alkaloids	ROS scavenging confirmed by HPLC-DPPH guided isolation	[18,26]
*Dracocephalum tanguticum* Maxim.	Flavonoids	Phenolic compounds	Reduction in oxidative stress and protective cellular effects	[19]
*Dracocephalum foetidum* Bunge	Monoterpene glycosides, phenylpropanoids	Terpenoids, phenylpropanoids	Modulation of oxidative stress pathways	[68]
*Dracocephalum rupestre* Hance	Flavonoids, phenolic compounds	Flavonoids, phenolic acids	Antioxidant activity associated with functional beverage (Maojian tea)	[20,28]
*Dracocephalum komarovi* Lipsky	Monoterpene glucosides, quinone derivatives	Terpenoids, quinones	Potential antioxidant activity via redox modulation mechanisms	[186,187,189]
*Dracocephalum forrestii* W.W.Sm.	Rosmarinic acid, salvianolic acid B	Phenolic acids	Strong antioxidant activity related to phenolic accumulation	[101,190]

**Table 2:** Antioxidant activity reported in different *Dracocephalum* species.

Species	Plant Material/Extract	Main Compounds Involved	Antioxidant Evaluation Method	References
*Dracocephalum moldavica* L.	Polyphenolic extract (aerial parts)	Rosmarinic acid, luteolin, apigenin derivatives	Radical scavenging assays	[41]
*Dracocephalum moldavica* L.	Seed extracts	Phenolic compounds, flavonoids	DPPH, FRAP, TPC	[21]
*Dracocephalum moldavica* L.	Herbal tea	Flavonoids, phenolic acids	Metabolic and antioxidant evaluation	[43,125]
*Dracocephalum kotschyi* Boiss.	Aerial parts	Xanthomicrol, luteolin, apigenin	Antioxidant assays	[13,23]
*Dracocephalum heterophyllum* Benth.	Aerial parts	Phenolic alkaloids, flavonoids	HPLC-DPPH guided isolation	[18,26]
*Dracocephalum tanguticum* Maxim.	Flavonoid fractions	Flavonoids	Cellular oxidative stress models	[19]
*Dracocephalum rupestre Hance*	Maojian tea	Phenolic compounds, flavonoids	Metabolomic profiling, antioxidant assays	[20,28]
*Dracocephalum komarovi* Lipsky	Aerial parts/isolated compounds	Monoterpene glucosides, quinones	Redox-related and anti-inflammatory assays	[186,187,188,189]
*Dracocephalum forrestii* W.W.Sm.	In vitro cultures	Rosmarinic acid, salvianolic acid B	Phenolic content & antioxidant evaluation	[101,190]
*Dracocephalum foetidum* Bunge	Aerial parts	Monoterpene glycosides, phenylpropanoids	Phytochemical analysis & inferred antioxidant activity	[68]
*Dracocephalum officinale* L.	Aerial parts/80% methanolic extract/essential oil	Rosmarinic acid, caffeic acid, pinocamphone, isopinocamphone, β-pinene	DPPH free radical scavenging assay; HPLC analysis of phenolic acids; GC–MS analysis of volatile compounds	[198]

## Data Availability

The original contributions presented in this study are included in the article. Further inquiries can be directed to the corresponding author.

## Abbreviations

The following abbreviations are used in this manuscript:

ABTS	2,2′-azino-bis(3-ethylbenzothiazoline-6-sulfonic acid)
DPPH	2,2-diphenyl-1-picrylhydrazyl
FRAP	ferric reducing antioxidant power
DNA	deoxyribonucleic acid
RNA	ribonucleic acid
ROS	reactive oxygen species
TPC	total phenolic content
TFC	total flavonoid content
C10	10 carbon atoms
C15	15 carbon atoms
C6-C3-C6	two aromatic rings linked by a three-carbon heterocyclic unit
HPLC	high-performance liquid chromatography
LC–MS	liquid chromatography–mass spectrometry
GC–MS	gas chromatography–mass spectrometry
UPLC	ultra-performance liquid chromatography
UPLC–MS	ultra-performance liquid chromatography–mass spectrometry
EO	essential oil
NO	nitric oxide
Nrf2	nuclear factor erythroid 2-related factor 2
Keap1	Kelch-like ECH-associated protein 1
NASH	nonalcoholic steatohepatitis
MASH	metabolic dysfunction-associated steatohepatitis
UPLC-Q-TOF-MS/MS	ultra-performance liquid chromatography–quadrupole time-of-flight mass spectrometry/mass spectrometry
CCl4	carbon tetrachloride
NF-κB	nuclear factor kappa B
TNF-α	tumor necrosis factor alpha
IL-6	interleukin 6
p38 MAPK	p38 mitogen-activated protein kinase
cAMP	cyclic adenosine monophosphate
ARE	antioxidant response element
HO-1	heme oxygenase-1
NQO1	NAD(P)H quinone oxidoreductase 1
γ-GCS	gamma-glutamylcysteine synthetase
IBS	irritable bowel syndrome
IBS-D	diarrhea-predominant irritable bowel syndrome
IBS-QOL	irritable bowel syndrome quality of life
PSQI	Pittsburgh Sleep Quality Index
GABA	gamma-aminobutyric acid
RCT	randomized controlled trial
BGN	biglycan
COL3A1	collagen type III alpha 1 chain
COL6A1	collagen type VI alpha 1 chain
COL16A1	collagen type XVI alpha 1 chain

## References

[1] Jäntschi L., Bolboacă S.D. (2014). Rarefaction on natural compound extracts diversity among genus. J. Comput. Sci..

[2] Weremczuk-Jeżyna I., Grzegorczyk-Karolak I. (2025). A comprehensive review of the phenolic compounds in *Dracocephalum* genus (Lamiaceae) related to traditional uses of the species and their biological activities. Molecules.

[3] Zhan M., Ma M., Mo X., Zhang Y., Li T., Yang Y., Dong L. (2024). *Dracocephalum moldavica* L.: An updated comprehensive review of its botany, traditional uses, phytochemistry, pharmacology, and application aspects. Fitoterapia.

[4] Chen Y.-P., Turdimatovich T.O., Nuraliev M.S., Lazarevic P., Drew B.T., Xiang C.-L. (2022). Phylogeny and biogeography of the northern temperate genus *Dracocephalum* s.l. (Lamiaceae). Cladistics.

[5] Liu H., Feng X., Zhao Y., Lv G., Zhang C., Aruhan, Damba T.-A., Zhang N., Hao D., Li M. (2024). Pharmacophylogenetic relationships of genus *Dracocephalum* and its related genera based on multifaceted analysis. Front. Pharmacol..

[6] Heydari P., Yavari M., Adibi P., Asghari G., Ghanadian S.-M., Dida G.O., Khamesipour F. (2019). Medicinal properties and active constituents of *Dracocephalum kotschyi* and its significance in Iran: A systematic review. Evid.-Based Complement. Altern. Med..

[7] Xu M.-L., Gao H.-M., Zhang Y.-X., Li Z.-J., Ding Y., Wang Q.-R., Huo S.-X., Feng W.-H., Kang Y.-T., Chen L.-M. (2024). Qualitative and quantitative analysis of chemical components of *Dracocephalum moldavica* based on UPLC-Q-TOF-MS/MS and UPLC. Zhongguo Zhong Yao Za Zhi.

[8] Chitiala R.D., Lungu I.I., Mitran A.-M., Mita-Baciu I., Brinza I., Mircea C., Nistor A., Hancianu M., Iliescu R., Hritcu L. (2025). Eco-Friendly Synthesis of Silver Nanoparticles Using Lespedeza capitata Extract: Antioxidant and Anti-Inflammatory Properties in Zebrafish (*Danio rerio*). Int. J. Mol. Sci..

[9] Chen Y.-C., Lei L.-J., Xiao T.-M., Xu Y.-N., Xing J.-G., Si S.-Y., Zheng R.-F., Chen M.-H. (2023). Moldavica acid A, a new salicylic acid derivative from *Dracocephalum moldavica*. J. Asian Nat. Prod. Res..

[10] Sabrin M.S., Selenge E., Takeda Y., Batkhuu J., Ogawa H., Jamsransuren D., Suganuma K., Murata T. (2021). Isolation and evaluation of virucidal activities of flavanone glycosides and rosmarinic acid derivatives from *Dracocephalum* spp. against feline calicivirus. Phytochemistry.

[11] Lee S.-E., Okhlopkova Z., Lim C., Cho S. (2020). *Dracocephalum palmatum* Stephan extract induces apoptosis in human prostate cancer cells via the caspase-8-mediated extrinsic pathway. Chin. J. Nat. Med..

[12] Martínez-Vázquez M., Estrada-Reyes R., Martínez-Laurrabaquio A., López-Rubalcava C., Heinze G. (2012). Neuropharmacological study of *Dracocephalum moldavica* L. (Lamiaceae) in mice: Sedative effect and chemical analysis of an aqueous extract. J. Ethnopharmacol..

[13] Jahaniani F., Ebrahimi S.A., Rahbar-Roshandel N., Mahmoudian M. (2005). Xanthomicrol is the main cytotoxic component of *Dracocephalum kotschyii* and a potential anti-cancer agent. Phytochemistry.

[14] Sheykhjan M.G., Fazlara A., Hojjati M., Alizadeh Behbahani B. (2025). Active food packaging strategies: Promoting mutton quality using Lepidium sativum coatings and nanoemulsions of *Dracocephalum moldavica* essential oil. Appl. Food Res..

[15] Zhang B., Ding R., He X., Li Y., He Z., Fang J., Guo M., Wang X., Zhao Z., Song X. (2024). *Dracocephalum moldavica* L.-derived carbon dots for effective periodontitis treatment via reprogramming energy metabolism in macrophages. Chem. Eng. J..

[16] Borghei S.F., Azizi A., Pourhosseini S.H., Rahimi-Rizi M. (2024). Characterization of dragonhead (*Dracocephalum moldavica* L.) landraces: Genetic, chemotypic, and agro-morphologic perspectives. J. Appl. Res. Med. Aromat. Plants.

[17] Shafiee-Kandjani A., Khalili M., Malek A., Farhang S., Ranjbari Y., Khalili Y. (2023). The therapeutic effect of the extracts of *Lavandula angustifolia* and *Dracocephalum ruyschiana* besides sertraline on patients with mild to moderate depression: A double-blind controlled trial. Phytomed. Plus.

[18] Wang L., Wang S., Yang S., Guo X., Lou H., Ren D. (2012). Phenolic alkaloids from the aerial parts of *Dracocephalum heterophyllum*. Phytochemistry.

[19] Wang S.-Q., Han X.-Z., Li X., Ren D.-M., Wang X.-N., Lou H.-X. (2010). Flavonoids from *Dracocephalum tanguticum* and their cardioprotective effects against doxorubicin-induced toxicity in H9c2 cells. Bioorg. Med. Chem. Lett..

[20] Lungu I., Huzum B., Humulescu I.A., Cioanca O., Morariu D., Șerban I.L., Hancianu M. (2020). Flavonoids as promising therapeutic and dietary agents. Med.-Surg. J..

[21] Song E., Choi J., Gwon H., Lee K.-Y., Choi S.-G., Islam M.A., Chun J., Hwang J. (2021). Phytochemical profile and antioxidant activity of *Dracocephalum moldavica* L. seed extracts using different extraction methods. Food Chem..

[22] Pouresmaeil M., Sabzi-Nojadeh M., Movafeghi A., Nezhadasad Aghbash B., Kosari-Nasab M., Zengin G., Maggi F. (2022). Phytotoxic activity of Moldavian dragonhead (*Dracocephalum moldavica* L.) essential oil and its possible use as bio-herbicide. Process Biochem..

[23] Moradi H., Ghavam M., Tavili A. (2020). Study of antioxidant activity and some herbal compounds of *Dracocephalum kotschyi* Boiss. in different ages of growth. Biotechnol. Rep..

[24] Kamali H., Khodaverdi E., Hadizadeh F., Ghaziaskar S.H. (2016). Optimization of phenolic and flavonoid content and antioxidants capacity of pressurized liquid extraction from *Dracocephalum kotschyi* via circumscribed central composite. J. Supercrit. Fluids.

[25] Foroozandeh E., Asadi-Gharneh H.A. (2021). *Dracocephalum kotschyi* Boiss.: An Iranian endemic medicinal plant; A review. J. Med. Herbs.

[26] Dang J., Zhang L., Shao Y., Mei L., Liu Z., Yue H., Wang Q., Tao Y. (2018). Preparative isolation of antioxidative compounds from *Dracocephalum heterophyllum* using off-line two-dimensional reversed-phase liquid chromatography/hydrophilic interaction chromatography guided by on-line HPLC-DPPH assay. J. Chromatogr. B.

[27] Hu L.-J., Zeng L., Ma E.-G., Yang C., Wu H.-Y., Li G.-P. (2020). Dratanguticumides G and H, two new glucosides from *Dracocephalum tanguticum* Maxim relax vessels via NO pathway. Phytochem. Lett..

[28] Zhang C., Su J., Wang J., Zhao Z. (2024). Identification of volatile and odor-active compounds in Maojian herbal tea (*Dracocephalum rupestre* Hance). J. Food Compos. Anal..

[29] Ren D.-M., Qu Z., Wang X.-N., Shi J., Lou H.-X. (2008). Simultaneous determination of nine major active compounds in *Dracocephalum rupestre* by HPLC. J. Pharm. Biomed. Anal..

[30] Hazrati S., Habibzadeh F., Davatgar-Iranizad A., Ebadi M.-T. (2026). Changes in essential oil content and composition of *Dracocephalum multicaule* at different phenological stages: Determination of balsamic time. Biochem. Syst. Ecol..

[31] Esmaeili M.A., Sonboli A., Mirjalili M.H. (2014). Oxidative stress protective effect of *Dracocephalum multicaule* essential oil against human cancer cell line. Nat. Prod. Res..

[32] Ángeles-López G.E., Hernández-Ruíz A., González-Trujano M.E., Cristians S., Ovalle-Magallanes B., Ventura-Martínez R. (2024). Pharmacological disadvantages in the spasmolytic effects by using the mixture known as “three toronjiles” in folk medicine. J. Ethnopharmacol..

[33] Joulaei Hossein Abadi S., Asadi-Gharneh H.A., Khalili F. (2026). Gamma-irradiated sodium alginate enhances growth, antioxidant defense, and citral-rich essential oil in *Dracocephalum kotschyi*. Ind. Crops Prod..

[34] Piao Y., Wu Z., Zhang Y., Wu Q., Piao J., Gao M., Ye F., Zhang C., Jin L. (2026). Chemical constituents of *Parasenecio auriculatus* (DC.) H. Koyama and their chemotaxonomic significance. Biochem. Syst. Ecol..

[35] Ren D.-M., Guo H.-F., Wang S.-Q., Lou H.-X. (2007). Separation and structure determination of two diastereomeric pairs of enantiomers from *Dracocephalum rupestre* by high-performance liquid chromatography with circular dichroism detection. J. Chromatogr. A.

[36] Talebi M., Ghassempour A., Feizi A., Ayatollahi S.A., Faizi M. (2024). Promising neuroprotective effects of *Dracocephalum* species: Mechanistic perspectives. Tradit. Integr. Med..

[37] Scotti L., Singla R.K., Ishiki H.M., Mendonça Júnior F.J.B., da Silva M.S., Barbosa Filho J.M., Scotti M.T. (2016). Recent advancement in natural hyaluronidase inhibitors. Curr. Top. Med. Chem..

[38] Fattahi A., Shakeri A., Tayarani-Najaran Z., Kharbach M., Segers K., Vander Heyden Y., Taghizadeh S.F., Rahmani H., Asili J. (2021). UPLC–PDA–ESI–QTOF–MS/MS and GC–MS analysis of Iranian *Dracocephalum moldavica* L.. Food Sci. Nutr..

[39] Balasundram N., Sundram K., Samman S. (2006). Phenolic compounds in plants and agri-industrial by-products: Antioxidant activity, occurrence, and potential uses. Food Chem..

[40] Simea Ș., Ielciu I., Hanganu D., Niculae M., Pall E., Burtescu R.F., Olah N.-K., Cenariu M., Oniga I., Benedec D. (2023). Evaluation of the cytotoxic, antioxidative and antimicrobial effects of *Dracocephalum moldavica* L. cultivars. Molecules.

[41] Aprotosoaie A.C., Mihai C.T., Vochita G., Rotinberg P., Trifan A., Luca S.V., Petreus T., Gille E., Miron A. (2016). Antigenotoxic and antioxidant activities of a polyphenolic extract from European *Dracocephalum moldavica* L.. Ind. Crops Prod..

[42] Sultan A., Bahang, Aisa H.A., Eshbakova K.A. (2008). Flavonoids from *Dracocephalum moldavica*. Chem. Nat. Compd..

[43] Nazemisalman B., Taheri S.S., Heydari F., Yazdinezhad A., Haghi F., Shabouei Jam M., Basir Shabestari S. (2024). Comparison of *Dracocephalum moldavica* essential oil with chlorhexidine on cariogenic bacteria. J. Dent. Shiraz Univ. Med. Sci..

[44] Kakasy A.Z., Lemberkovics E., Simándi B., Lelik L., Héthelyi É., Antal I., Szöke E. (2006). Comparative study of traditional essential oil and supercritical fluid extracts of Moldavian dragonhead (*Dracocephalum moldavica* L.). Flavour Fragr. J..

[45] Saeidnia S., Gohari A.R., Uchiyama N., Ito M., Honda G., Kiuchi F. (2004). Two new monoterpene glycosides and trypanocidal terpenoids from *Dracocephalum kotschyi*. Chem. Pharm. Bull..

[46] Chelan Z.A., Amini R., Mohammadi Nasab A.D. (2023). Essential Oil Yield and Compositions of *Dracocephalum moldavica* L. in Intercropping with Fenugreek, Inoculation with Mycorrhizal Fungi and Bacteria. Sci. Rep..

[47] Dastmalchi K., Dorman H.J.D., Kosar M., Hiltunen R. (2007). Chemical composition and in vitro antioxidant evaluation of a water-soluble Moldavian balm (*Dracocephalum moldavica* L.) extract. LWT-Food Sci. Technol..

[48] Bakkali F., Averbeck S., Averbeck D., Idaomar M. (2008). Biological effects of essential oils—A review. Food Chem. Toxicol..

[49] Raut J.S., Karuppayil S.M. (2014). A status review on the medicinal properties of essential oils. Ind. Crops Prod..

[50] Amini R., Ebrahimi A., Dabbagh Mohammadi Nasab A. (2020). Moldavian balm (*Dracocephalum moldavica* L.) essential oil content and composition as affected by sustainable weed management treatments. Ind. Crops Prod..

[51] Nasiri Y., Zandi H., Morshedloo M.R. (2018). Effect of Salicylic Acid and Ascorbic Acid on Essential Oil Content and Composition of Dragonhead (*Dracocephalum moldavica* L.) under Organic Farming. J. Essent. Oil Bear. Plants.

[52] Yang L.-N., Xing J.-G., He C.-H., Wu T. (2014). The phenolic compounds from *Dracocephalum moldavica* L.. Biochem. Syst. Ecol..

[53] Rudy S., Dziki D., Biernacka B., Krzykowski A., Rudy M., Gawlik-Dziki U., Kachel M. (2020). Drying Characteristics of *Dracocephalum moldavica* Leaves: Drying Kinetics and Physicochemical Properties. Processes.

[54] Atazhanova G., Sabiyeva A., Akhmetova S., Smagulov M., Medeshova A., Sarsembayeva S., Sarsembayeva A., Aldabayeva U., Kurmantayeva G. (2023). Component Composition and Antimicrobial Activity of *Dracocephalum nutans* L. Essential Oil. Res. J. Pharm. Technol..

[55] Golmakani M.-T., Rezaei K. (2008). Comparison of microwave-assisted hydrodistillation with traditional hydrodistillation method in the extraction of essential oils from *Thymus vulgaris* L.. Food Chem..

[56] Avagyan A., Martirosyan G., Brindza J., Sargsyan G., Vardanian I., Harutyunyan Z., Balayan R., Harutyunyan M., Hovhannisyan M., Tadevosyan L. (2025). Antimicrobial activity of essential oils from introduced varieties of *Dracocephalum moldavica* and *Hyssopus officinalis*. Funct. Food Sci..

[57] Aćimović M., Šovljanski O., Šeregelj V., Pezo L., Zheljazkov V.D., Ljujić J., Tomić A., Ćetković G., Čanadanović-Brunet J., Miljković A. (2022). Chemical composition, antioxidant, and antimicrobial activity of *Dracocephalum moldavica* L. essential oil and hydrolate. Plants.

[58] Aćimović M., Stanković J., Cvetković M., Todosijević M., Rat M. (2019). The chemical composition of the essential oil of *Dracocephalum moldavica* L. from Vojvodina Province (Serbia). Biol. Nyssana.

[59] Said-Al Ahl H.A., Sabra A.S., El Gendy A.N.G., Aziz E.E., Tkachenko K. (2015). Changes in content and chemical composition of *Dracocephalum moldavica* L. essential oil at different harvest dates. J. Med. Plants Stud..

[60] Maham M., Akbari H., Delazar A. (2013). Chemical composition and antinociceptive effect of the essential oil of *Dracocephalum moldavica* L.. Pharm. Sci..

[61] Teymoorian M., Moghimi R., Hosseinzadeh R., Zandi F., Lakouraj M.M. (2024). Fabrication the emulsion-based edible film containing *Dracocephalum kotschyi* boiss essential oil using chitosan–gelatin composite for grape preservation. Carbohydr. Polym. Technol. Appl..

[62] Maghsoudi K., Partovi R., Alian Samakkhah S. (2025). Evaluation of the Effect of *Dracocephalum moldavica* Mucilage and Nanoemulsion of *Satureja hortensis* Essential Oil Edible Coating on Microbial, Chemical, and Organoleptic Properties of Rainbow Trout Fish Fillet at Refrigerator Temperature. Food Sci. Nutr..

[63] Hyldgaard M., Mygind T., Meyer R.L. (2012). Essential oils in food preservation: Mode of action, synergies, and interactions with food matrix components. Front. Microbiol..

[64] Tongnuanchan P., Benjakul S. (2014). Essential oils: Extraction, bioactivities, and their uses for food preservation. J. Food Sci..

[65] Burt S. (2004). Essential oils: Their antibacterial properties and potential applications in foods—A review. Int. J. Food Microbiol..

[66] Bassolé I.H.N., Juliani H.R. (2012). Essential oils in combination and their antimicrobial properties. Molecules.

[67] Yousefzadeh S., Daryai F., Mokhtassi-Bidgoli A., Hazrati S., Yousefzadeh T., Mohammadi K. (2018). Morphological, essential oil and biochemical variation of *Dracocephalum moldavica* L. populations. J. Appl. Res. Med. Aromat. Plants.

[68] Selenge E., Murata T., Tanaka S., Sasaki K., Batkhuu J., Yoshizaki F. (2014). Monoterpene glycosides, phenylpropanoids, and acacetin glycosides from *Dracocephalum foetidum*. Phytochemistry.

[69] Weremczuk-Jeżyna I., Grzegorczyk-Karolak I., Frydrych B., Hnatuszko-Konka K., Gerszberg A., Wysokińska H. (2017). Rosmarinic Acid Accumulation and Antioxidant Potential of *Dracocephalum moldavica* L. Cell Suspension Culture. Not. Bot. Horti Agrobot..

[70] Sun X., Ge N., Liang Q., Wang Q., Yu H., Jin M. (2025). Effect of the Total Flavonoids of *Dracocephalum moldavica* L. on Metabolic Associated Fatty Liver Disease in Rats. Front. Pharmacol..

[71] Dawuti A., Sun S., Wang R., Gong D., Yuan T., Zhang L., Yang S., Xing J., Zheng R., Lu Y. (2022). Systems Pharmacology-Based Strategy to Investigate Pharmacological Mechanisms of Total Flavonoids in *Dracocephalum moldavica* on Chronic Heart Failure. Int. J. Mol. Sci..

[72] Kumar S., Pandey A.K. (2013). Chemistry and Biological Activities of Flavonoids: An Overview. Sci. World J..

[73] Gao T., Wu L., Ma Y., Zhai W., Zhang L., Cong W., Cai Z., Cui C., Li L. (2025). Pickering emulsion loaded with total flavonoids from *Dracocephalum moldavica* L. potentially promotes angiogenesis in the ischemic penumbra after cerebral ischemia reperfusion. Front. Bioeng. Biotechnol..

[74] Chen S., Wang X., Cheng Y., Gao H., Chen X. (2023). A Review of Classification, Biosynthesis, Biological Activities and Potential Applications of Flavonoids. Molecules.

[75] Abd El-Aleem M.I., Abd El-Rahman A.A., El-Hadary A.E. Identification and Characterization of Antioxidant and Bioactive Components of *Mirabilis jalapa* and *Dracocephalum moldavica* L. Plants. Proceedings of the 5th International Conference on Biotechnology Applications in Agriculture (ICBAA).

[76] Akanda M.R., Uddin M.N., Kim I.-S., Ahn D., Tae H.-J., Park B.-Y. (2019). The Biological and Pharmacological Roles of Polyphenol Flavonoid Tilianin. Eur. J. Pharmacol..

[77] Jang J.Y., Kim D., Im E., Lee N.K., Kim N.D. (2025). Molecular mechanism discovery of acacetin against cancers: Insights from network pharmacology and molecular docking. Int. J. Mol. Sci..

[78] Abdel-Rasol M.A., Abbas R.A.R., El-Sayed W.M. (2025). Tilianin: Pharmacological potential, mechanisms of action, and future perspectives in traditional and modern medicine. Chin. Med..

[79] Weremczuk-Jeżyna I., Gonciarz W., Grzegorczyk-Karolak I. (2023). Antioxidant and Anti-Inflammatory Activities of Phenolic Acid-Rich Extract from Hairy Roots of *Dracocephalum moldavica*. Molecules.

[80] Sun M., Guo M., He Z., Luo Y., Yang H., Zhang Y., Li J., Cao W., Huang C., Wang L. (2025). Enhancing oral bioavailability and reducing gastrointestinal toxicity of tilianin via folic acid-modified nanocrystal liposomes. J. Drug Deliv. Sci. Technol..

[81] Oniszczuk T., Kasprzak-Drozd K., Olech M., Wójtowicz A., Nowak R., Rusinek R., Szponar J., Combrzyński M., Oniszczuk A. (2021). The Impact of Formulation on the Content of Phenolic Compounds in Snacks Enriched with *Dracocephalum moldavica* L. Seeds: Introduction to Receiving a New Functional Food Product. Molecules.

[82] Okhlopkova Z.M., Razgonova M.P., Rozhina Z.G., Egorova P.S., Golokhvast K.S. (2023). *Dracocephalum jacutense* Peschkova from Yakutia: Extraction and Mass Spectrometric Characterization of 128 Chemical Compounds. Molecules.

[83] Jiang J., Yuan X., Wang T., Chen H., Zhao H., Yan X., Wang Z., Sun X., Zheng Q. (2014). Antioxidative and Cardioprotective Effects of Total Flavonoids Extracted from *Dracocephalum moldavica* L. against Acute Ischemia/Reperfusion-Induced Myocardial Injury in Isolated Rat Heart. Cardiovasc. Toxicol..

[84] Gao J., Wang Z., Chen D., Peng J., Xie D., Lin Z., Lin Z., Dai W. (2022). Metabolomic Characterization of the Chemical Compositions of *Dracocephalum rupestre* Hance. Food Res. Int..

[85] Krishnaiah D., Sarbatly R., Nithyanandam R. (2011). A review of the antioxidant potential of medicinal plant species. Food Bioprod. Process..

[86] Dolkun A., Muhammad T., Gao J., Chen Y., Zhang Y., Zhi D., Zhang S. (2024). Batch adsorption kinetic and thermodynamic studies of flavonoids from *Dracocephalum moldavia* via flow injection online measurement. Sep. Purif. Technol..

[87] Adelnina N., Mosavi Dehmordi L., Chermahini M.A., Sori M. (2024). Antioxidant and immunological properties of *Dracocephalum moldavica* extract in *Penaeus vannamei*. Nat. Prod. Commun..

[88] Dini S., Chen Q., Fatemi F., Asri Y. (2022). Phytochemical and Biological Activities of Some Iranian Medicinal Plants. Pharm. Biol..

[89] Zhu C.-S., Liu K., Wang J.-L., Li J.-F., Liu M.-F., Hao N., Lin Y.-X., Xiao Z.-F. (2018). Antioxidant activities and hepatoprotective potential of *Dracocephalum rupestre* Hance extract against CCl4-induced hepatotoxicity in Kunming mice. J. Food Biochem..

[90] Han X., Gao S., Cheng Y., Sun Y., Liu W., Tang L., Ren D. (2012). Protective effect of naringenin-7-O-glucoside against oxidative stress induced by doxorubicin in H9c2 cardiomyocytes. BioSci. Trends.

[91] Sonboli A., Mojarrad M., Gholipour A., Nejad Ebrahimi S., Arman M. (2008). Biological Activity and Composition of the Essential Oil of *Dracocephalum moldavica* L. Grown in Iran. Nat. Prod. Commun..

[92] Li S., Li Q., Zhang N., Li M. (2025). Hypolipidemic activity and mechanism of active components of *Dracocephalum moldavica L.* tea. Food Sci. Hum. Wellness.

[93] Shahidi F., Ambigaipalan P. (2015). Phenolics and polyphenolics in foods, beverages and spices: Antioxidant activity and health effects—A review. J. Funct. Foods.

[94] Tsao R. (2010). Chemistry and biochemistry of dietary polyphenols. Nutrients.

[95] Rice-Evans C.A., Miller N.J., Paganga G. (1996). Structure–antioxidant activity relationships of flavonoids and phenolic acids. Free Radic. Biol. Med..

[96] Dai J., Mumper R.J. (2010). Plant phenolics: Extraction, analysis and their antioxidant and anticancer properties. Molecules.

[97] Pandey K.B., Rizvi S.I. (2009). Plant polyphenols as dietary antioxidants in human health and disease. Oxid. Med. Cell. Longev..

[98] Croft K.D. (1998). The chemistry and biological effects of flavonoids and phenolic acids. Ann. N. Y. Acad. Sci..

[99] Heleno S.A., Martins A., Queiroz M.J.R.P., Ferreira I.C.F.R. (2015). Bioactivity of phenolic acids: Metabolites versus parent compounds: A review. Food Chem..

[100] Scalbert A., Johnson I.T., Saltmarsh M. (2005). Polyphenols: Antioxidants and beyond. Am. J. Clin. Nutr..

[101] Weremczuk-Jeżyna I., Skała E., Olszewska M.A., Kiss A.K., Balcerczak E., Wysokińska H., Kicel A. (2016). The identification and quantitative determination of rosmarinic acid and salvianolic acid B in hairy root cultures of *Dracocephalum forrestii* W.W. Smith. Ind. Crops Prod..

[102] Petersen M., Simmonds M.S.J. (2003). Rosmarinic acid. Phytochemistry.

[103] Amoah S.K.S., Sandjo L.P., Kratz J.M., Biavatti M.W. (2016). Rosmarinic Acid—Pharmaceutical and Clinical Aspects. Planta Med..

[104] Varasteh Khanlari N., Kiarostami K., Hosseinzadeh Namin M., Abdoli M., Karamian R. (2025). Rosmarinic acid content and antioxidant capacity in *Dracocephalum moldavica* hairy roots affected by iron and copper nanoparticles. Plant Cell Tissue Organ Cult..

[105] Bravo L. (1998). Polyphenols: Chemistry, dietary sources, metabolism, and nutritional significance. Nutr. Rev..

[106] Hidalgo M., Sánchez-Moreno C., de Pascual-Teresa S. (2010). Flavonoid–Flavonoid Interaction and Its Effect on Their Antioxidant Activity. Food Chem..

[107] Khoddami A., Wilkes M.A., Roberts T.H. (2013). Techniques for analysis of plant phenolic compounds. Molecules.

[108] Golparvar A.R., Hadipanah A., Gheisari M.M., Khaliliazar R. (2016). Chemical constituents of essential oil of Dracocephalum *moldavica* L. and *Dracocephalum kotschyi* Boiss. from Iran. Acta Agric. Slov..

[109] Naczk M., Shahidi F. (2004). Extraction and analysis of phenolics in food. J. Chromatogr. A.

[110] Manach C., Scalbert A., Morand C., Rémésy C., Jiménez L. (2004). Polyphenols: Food sources and bioavailability. Am. J. Clin. Nutr..

[111] Ignat I., Volf I., Popa V.I. (2011). A critical review of methods for characterization of polyphenolic compounds in fruits and vegetables. Food Chem..

[112] Zhang H., Wang S., Liu Q., Zheng H., Liu X., Wang X., Shen T., Ren D. (2021). Dracomolphin A–E, new lignans from *Dracocephalum moldavica*. Fitoterapia.

[113] Wang J., Sun J., Wang M., Cui H., Zhou W., Li G. (2022). Chemical constituents from *Dracocephalum moldavica* L. and their chemotaxonomic significance. Biochem. Syst. Ecol..

[114] Zhang C., Li H., Yun T., Fu Y., Liu C., Gong B., Neng B. (2008). Chemical composition, antimicrobial and antioxidant activities of the essential oil of Tibetan herbal medicine *Dracocephalum heterophyllum* Benth. Nat. Prod. Res..

[115] Povilaityte V., Cuvelier M.-E., Berset C. (2001). Antioxidant properties of Moldavian dragonhead (*Dracocephalum moldavica* L.). J. Food Lipids.

[116] Yu H., Liu M., Liu Y., Qin L., Jin M., Wang Z. (2019). Antimicrobial activity and mechanism of action of *Dracocephalum moldavica* L. extracts against clinical isolates of Staphylococcus aureus. Front. Microbiol..

[117] Nikitina A.S., Popova O.I., Ushakova L.S., Chumakova V.V., Ivanova L.I. (2008). Studies of the essential oil of *Dracocephalum moldavica* cultivated in the Stavropol region. Pharm. Chem. J..

[118] Popova O.I., Nikitina A.S., Markova O.M. (2008). Studies of Iridoids from *Dracocephalum moldavica* Cultivated in the Stavropol Region. Pharm. Chem. J..

[119] Yousefzadeh S., Modarres-Sanavy S.A.M., Sefidkon F., Asgarzadeh A., Ghalavand A., Sadat-Asilan K. (2013). Effects of azocompost and urea on the herbage yield and contents and compositions of essential oils from two genotypes of dragonhead (*Dracocephalum moldavica* L.) in two regions of Iran. Food Chem..

[120] Jiang H., Zeng L., Dong X., Guo S., Xing J., Li Z., Liu R. (2020). Tilianin extracted from *Dracocephalum moldavica* L. induces intrinsic apoptosis and drives inflammatory microenvironment response on pharyngeal squamous carcinoma cells via regulating TLR4 signaling pathways. Front. Pharmacol..

[121] Postu P.A., Gorgan D.L., Cioanca O., Russ M., Mikkat S., Glocker M.O., Hritcu L. (2020). Memory-Enhancing Effects of *Origanum majorana* Essential Oil in an Alzheimer’s Amyloid beta1-42 Rat Model: A Molecular and Behavioral Study. Antioxidants.

[122] Tan M.-E., He C.-H., Jiang W., Zeng C., Yu N., Huang W., Gao Z.-G., Xing J.-G. (2017). Development of solid lipid nanoparticles containing total flavonoid extract from *Dracocephalum moldavica* L. and their therapeutic effect against myocardial ischemia–reperfusion injury in rats. Int. J. Nanomed..

[123] Komarov A., Naida N., Nugis E. (2021). Dynamic model of seed germination on the example of a genus *Dracocephalum L.* based on logistic function. Agraarteadus.

[124] Weremczuk-Jeżyna I., Grzegorczyk-Karolak I., Frydrych B., Królicka A., Wysokińska H. (2013). Hairy Roots of *Dracocephalum moldavica*: Rosmarinic Acid Content and Antioxidant Potential. Acta Physiol. Plant..

[125] Li Q., Li S., Chen A., Huang C., Chang J., Zhang N., Li M. (2026). *Dracocephalum moldavica* L. tea alleviates high-fat diet-induced hyperlipidemia in rats via gut microbiota and lipid metabolism. J. Future Foods.

[126] Beigomi M., Mohsenzadeh M., Salari A. (2018). Characterization of a novel biodegradable edible film obtained from *Dracocephalum moldavica* seed mucilage. Int. J. Biol. Macromol..

[127] Koohdar F., Sheidai M. (2019). Molecular investigation in few species of *Dracocephalum* in Iran: Species relationship, reticulation and divergence time. Ind. Crops Prod..

[128] Fattahi M., Nazeri V., Torras-Claveria L., Sefidkon F., Cusido R.M., Zamani Z., Palazon J. (2013). Identification and quantification of leaf surface flavonoids in wild-growing populations of *Dracocephalum kotschyi* by LC–DAD–ESI-MS. Food Chem..

[129] Sheidai M., Koohdar F., Mazinani J. (2024). Geographical and genetic clines in *Dracocephalum* kotschyi × *Dracocephalum oligadenium* hybrids: Landscape genetics and genocline analyses. BMC Plant Biol..

[130] Zamani S., Bakhshi D., Ebadi M.-T., Sahraroo A. (2025). Phytochemical profile and antioxidant activity of *Dracocephalum kotschyi* Boiss. affected by environmental conditions of cultivation regions. BMC Plant Biol..

[131] Nwachukwu K.C., Alsahafi S., Ndom H.U., Ugbogu O.C., Oyewole O.A. (2026). The effects of cobalt and titanium nanoparticles on plant health. Plant Nano Biol..

[132] Lungu I.L., Batir-Marin D., Panainte A., Mircea C., Tuchilus C., Stefanache A., Szasz F.A., Grigorie D., Robu S., Cioanca O. (2023). Catechin-Zinc-Complex: Synthesis, Characterization and Biological Activity Assessment. Farmacia.

[133] Javidnia K., Miri R., Kamalinejad M., Khoshneviszadeh M. (2006). Constituents of the volatile oils of *Dracocephalum kotschyi* Boiss. from Iran. J. Essent. Oil Res..

[134] Saeidnia S., Gohari A.R., Hadjiakhoondi A., Shafiee A. (2007). Bioactive compounds of the volatile oil of *Dracocephalum kotschyi*. Z. Naturforsch. C.

[135] Ashrafi B., Ramak P., Ezatpour B., Talei G.R. (2017). Investigation on chemical composition, antimicrobial, antioxidant, and cytotoxic properties of essential oil from *Dracocephalum kotschyi* Boiss. Afr. J. Tradit. Complement. Altern. Med..

[136] Jalaei Z., Fattahi M., Aramideh S. (2015). Allelopathic and insecticidal activities of essential oil of *Dracocephalum kotschyi* Boiss. from Iran: A new chemotype with highest limonene-10-al and limonene. Ind. Crops Prod..

[137] Fallah S., Mouguee S., Rostaei M., Adavi Z., Lorigooini Z. (2020). Chemical Compositions and Antioxidant Activity of Essential Oil of Wild and Cultivated *Dracocephalum kotschyi* Grown in Different Ecosystems: A Comparative Study. Ind. Crops Prod..

[138] Dorosti N., Jamshidi F. (2016). Plant-mediated gold nanoparticles by *Dracocephalum kotschyi* as anticholinesterase agent: Synthesis, characterization, and evaluation of anticancer and antibacterial activity. J. Appl. Biomed..

[139] Sodeifian G., Sajadian S.A., Saadati Ardestani N. (2017). Supercritical Fluid Extraction of Omega-3 from *Dracocephalum kotschyi* Seed Oil: Process Optimization and Oil Properties. J. Supercrit. Fluids.

[140] Fattahi M., Bonfill M., Fattahi B., Torras-Claveria L., Sefidkon F., Cusido R.M., Palazon J. (2016). Secondary metabolites profiling of *Dracocephalum kotschyi* Boiss at three phenological stages using uni- and multivariate methods. J. Appl. Res. Med. Aromat. Plants.

[141] Moridi Farimani M., Mirzania F., Sonboli A., Matloubi Moghaddam F. (2017). Chemical composition and antibacterial activity of *Dracocephalum kotschyi* essential oil obtained by microwave extraction and hydrodistillation. Int. J. Food Prop..

[142] Zamani S., Bakhshi D., Sahraroo A., Ebadi M.-T. (2023). Improvement of phytochemical and quality characteristics of *Dracocephalum kotschyi* by drying methods. Food Sci. Nutr..

[143] Sajjadi S.E., Atar A.M., Yektaian A. (1998). Antihyperlipidemic effect of hydroalcoholic extract, and polyphenolic fraction from *Dracocephalum kotschyi* Boiss. Pharm. Acta Helv..

[144] Kamali M., Khosroyar S., Kamali H., Ahmadzadeh Sani T., Mohammadi A. (2016). Phytochemical screening and evaluation of antioxidant activities of *Dracocephalum kotschyi* and determination of its luteolin content. Avicenna J. Phytomed..

[145] Kalantar K., Gholijani N., Mousaei N., Yazdani M., Amirghofran Z. (2018). Investigation of *Dracocephalum kotschyi* Plant Extract on the Effective Inflammatory Transcription Factors and Mediators in Activated Macrophages. Anti-Inflamm. Anti-Allergy Agents Med. Chem..

[146] Kazempour M., Shahangian S.S., Sariri R. (2024). *Dracocephalum kotschyi*: Inhibition of critical enzyme relevant to type-2 diabetes, essential oil composition, bactericidal and antioxidant activity. Casp. J. Environ. Sci..

[147] Sarvestani N.N., Khodagholi F., Ansari N., Farimani M.M. (2013). Involvement of p-CREB and phase II detoxifying enzyme system in neuroprotection mediated by the flavonoid calycopterin isolated from *Dracocephalum kotschyi*. Phytomedicine.

[148] Sadraei H., Ghanadian S.M., Moazeni S. (2019). Inhibitory effect of hydroalcoholic and flavonoids extracts of *Dracocephalum kotschyi*, and its components luteolin, apigenin and apigenin-4′-galactoside on intestinal transit in mice. J. Herbmed Pharmacol..

[149] Khamesipour F., Razavi S.M., Hejazi S.H., Ghanadian S.M. (2021). In vitro and in vivo anti-Toxoplasma activity of *Dracocephalum kotschyi* essential oil. Food Sci. Nutr..

[150] Mirzania F., Moridi Farimani M. (2018). Biochemical evaluation of antioxidant activity, phenol and flavonoid contents of *Dracocephalum kotschyi* Boiss extracts obtained with different solvents. Health Biotechnol. Biopharma.

[151] Shaabani M., Mousavi S.H., Azizi M., Jafari A.A. (2020). Cytotoxic and apoptogenic effects of *Dracocephalum kotschyi* Boiss., extracts against human glioblastoma U87 cells. Avicenna J. Phytomed..

[152] Sadraei H., Asghari G., Kasiri F. (2015). Antispasmodic effect of *Dracocephalum kotschyi* hydroalcoholic extract on rat ileum contraction. Res. Pharm. Sci..

[153] Heydari P., Ghanadian M., Asghari G., Azimi M., Babaeian M., Adibi P. (2023). A double-blind randomized clinical trial of *Dracocephalum kotschyi* Boiss. in the patients with diarrhea-predominant irritable bowel syndrome. Res. Pharm. Sci..

[154] Sadraei H., Ghanadian S.M., Asghari G., Gavahian A. (2019). Bronchodilator effect of apigenin and luteolin, two components of *Dracocephalum kotschyi* on isolated rabbit trachea. J. Herbmed. Pharmacol..

[155] Fattahi M., Nazeri V., Torras-Claveria L., Sefidkon F., Cusido R.M., Zamani Z., Palazon J. (2013). A New Biotechnological Source of Rosmarinic Acid and Surface Flavonoids: Hairy Root Cultures of *Dracocephalum kotschyi* Boiss. Ind. Crops Prod..

[156] Chahardoli A., Karimi N., Fattahi A., Salimikia I. (2019). Biological applications of phytosynthesized gold nanoparticles using leaf extract of *Dracocephalum kotschyi*. J. Biomed. Mater. Res. A.

[157] Chahardoli A., Qalekhani F., Shokoohinia Y., Fattahi A. (2020). Biological and catalytic activities of green synthesized silver nanoparticles from the leaf infusion of *Dracocephalum kotschyi* Boiss. Glob. Chall..

[158] Asghari G., Nasr Esfahani B., Paydar P. (2015). Evaluating the effect of *Dracocephalum kotschyi* methanol extract on *Mycobacterium tuberculosis*. Res. J. Pharmacogn..

[159] Haghighi Pak Z., Abbaspour H., Karimi N., Fattahi A. (2016). Eco-friendly synthesis and antimicrobial activity of silver nanoparticles using *Dracocephalum moldavica* seed extract. Appl. Sci..

[160] Hesari M., Mohammadi P., Khademi F., Shackebaei D., Momtaz S., Moasefi N., Farzaei M.H., Abdollahi M. (2021). Current advances in the use of nanophytomedicine therapies for human cardiovascular diseases. Int. J. Nanomed..

[161] Sonboli A., Mirzania F., Gholipour A. (2019). Essential oil composition of *Dracocephalum kotschyi* Boiss. from Iran. Nat. Prod. Res..

[162] Lv Y., Wang Z., Wang Q., Dang J. (2022). Medium- and high-pressure integrated chromatographic strategies for the isolation and purification of free radical inhibitors from *Dracocephalum heterophyllum*. Separations.

[163] Lv Y., Wang Z., Wu Q., Fang Y., Wang Q., Li G., Dang J. (2022). Preparation and antioxidant activities of phenylethanoids from *Dracocephalum heterophyllum*. Separations.

[164] Numonov S., Sharopov F., Qureshi M.N., Gaforzoda L., Gulmurodov I., Khalilov Q., Setzer W.N., Habasi M., Aisa H.A. (2020). The Ursolic Acid-Rich Extract of *Dracocephalum heterophyllum* Benth. with Potent Antidiabetic and Cytotoxic Activities. Appl. Sci..

[165] Shi Q.-Q., Dang J., Wen H.-X., Yuan X., Tao Y.-D., Wang Q.-L. (2016). Anti-hepatitis, antioxidant activities and bioactive compounds of *Dracocephalum heterophyllum* extracts. Bot. Stud..

[166] Zheng W., Wang Q., Lu X., Shi Q., Zou J., Tao Y., Wang P. (2016). Protective Effects of *Dracocephalum heterophyllum* in ConA-Induced Acute Hepatitis. Mediat. Inflamm..

[167] Wang Y., Lai D., Geng Y., Shang P., Wang P. (2022). Therapeutic effects of *Dracocephalum heterophyllum* in collagen-induced arthritis. AAPS Open.

[168] Fang Y., Sun D., Li G., Lv Y., Li J., Wang Q., Dang J. (2022). Ethyl acetate extract of *Dracocephalum heterophyllum* Benth ameliorates nonalcoholic steatohepatitis and fibrosis via regulating bile acid metabolism, oxidative stress and inhibiting inflammation. Separations.

[169] Lv Y., Li C., Wang Z., Wang Q., Li G., Dang J. (2022). Preparative isolation of antioxidative furanocoumarins from *Dracocephalum heterophyllum* and their potential action target. J. Sep. Sci..

[170] Li C., Dang J., Lv Y., Fang Y., Ma C., Wang Q., Li G. (2022). The isolation and preparation of samwinol from *Dracocephalum heterophyllum* and prevention on Aβ25–35-induced neuroinflammation in PC-12 cells. Int. J. Mol. Sci..

[171] Stappen I., Wanner J., Tabanca N., Wedge D.E., Ali A., Kaul V.K., Lal B., Jaitak V., Gochev V.K., Schmidt E. (2015). Chemical composition and biological activity of essential oils of *Dracocephalum heterophyllum* and *Hyssopus officinalis* from Western Himalaya. Nat. Prod. Commun..

[172] Chander R., Maurya A.K., Kumar K., Kumari S., Kumar R., Agnihotri V.K. (2023). In vitro antidiabetic and antimicrobial activity of *Dracocephalum heterophyllum* Benth. essential oil from different sites of North-western Himalayas India. Nat. Prod. Res..

[173] Jiang H., Zhang C., He W. (2018). The effects of *Dracocephalum heterophyllum* Benth flavonoid on hypertrophic cardiomyocytes induced by angiotensin II in rats. Med. Sci. Monit..

[174] Guo M., Gu L., Hui H., Li X., Jin L. (2022). Extracts of *Dracocephalum tanguticum* Maxim ameliorate acute alcoholic liver disease via regulating transcription factors in mice. Front. Pharmacol..

[175] Li H., Fu Y., Sun H., Zhang Y., Lan X. (2017). Transcriptomic analyses reveal biosynthetic genes related to rosmarinic acid in *Dracocephalum tanguticum*. Sci. Rep..

[176] Wang S., Ren D., Xiang F., Wang X., Zhu C., Yuan H., Sun L., Lv B.-B., Sun X.-J., Lou H.-X. (2009). Dracotanosides A–D, Spermidine Glycosides from *Dracocephalum tanguticum*: Structure and Amide Rotational Barrier. J. Nat. Prod..

[177] Xu J.-X., Yang M., Deng K.-J., Zhou H. (2011). Antioxidant activities of *Dracocephalum tanguticum* maxim extract and its up-regulation on the expression of neurotrophic factors in a rat model of permanent focal cerebral ischemia. Am. J. Chin. Med..

[178] Wang T.-T., Li C.-P., Wei Y.-S., Dong Z.-Y., Meng F.-C., Chen M., Lan X.-Z. (2024). Dracotangusions A and B, two new sesquiterpenes from *Dracocephalum tanguticum* Maxim. with anti-inflammatory activity. Nat. Prod. Res..

[179] Wang X., Xu J., Yang M., Zhou H. (2011). Chloroform extract of Tibetan herbal medicine *Dracocephalum tanguticum* Maxim. inhibits proliferation of T98G glioblastomas cells by modulating Caspase-3 cleavage and expression of Bax and p21. J. Med. Plants Res..

[180] Ma E.-G., Wu H.-Y., Hu L.-J., Wei M., Mou L.-Y., Li G.-P. (2020). Three new phenylacetamide glycosides from *Dracocephalum tanguticum* Maxim and their anti-hyperglycemic activity. Nat. Prod. Res..

[181] Ren D.-M., Guo H.-F., Yu W.-T., Wang S.-Q., Ji M., Lou H.-X. (2008). Stereochemistry of flavonoidal alkaloids from *Dracocephalum rupestre*. Phytochemistry.

[182] Hu Q., Zhang D.D., Wang L., Lou H., Ren D. (2012). Eriodictyol-7-O-glucoside, a novel Nrf2 activator, confers protection against cisplatin-induced toxicity. Food Chem. Toxicol..

[183] Jing X., Shi H., Zhu X., Wei X., Ren M., Han M., Ren D., Lou H. (2015). Eriodictyol attenuates β-amyloid 25–35 peptide-induced oxidative cell death in primary cultured neurons by activation of Nrf2. Neurochem. Res..

[184] Lou H., Jing X., Ren D., Wei X., Zhang X. (2012). Eriodictyol protects against H2O2-induced neuron-like PC12 cell death through activation of Nrf2/ARE signaling pathway. Neurochem. Int..

[185] Wang C., Ma Z., Wang Z., Ming S., Ding Y., Zhou S., Qian H. (2021). Eriodictyol attenuates MCAO-induced brain injury and neurological deficits via reversing the autophagy dysfunction. Front. Syst. Neurosci..

[186] Uchiyama N., Kiuchi F., Ito M., Honda G., Takeda Y., Khodzhimatov O.K., Ashurmetov O.A. (2006). Trypanocidal constituents of *Dracocephalum komarovi*. Tetrahedron.

[187] Toshmatov Z.O., Li J., Eshbakova K.A., Tang D., Xin X., Aisa H.A. (2019). New monoterpene glucosides from *Dracocephalum komarovi* and their anti-inflammatory activity. Phytochem. Lett..

[188] Suto Y., Kaneko K., Yamagiwa N., Iwasaki G. (2010). A short and efficient asymmetric synthesis of komaroviquinone. Tetrahedron Lett..

[189] Suto Y., Sato M., Fujimori K., Kitabatake S., Okayama M., Ichikawa D., Matsushita M., Yamagiwa N., Iwasaki G., Kiuchi F. (2017). Synthesis and biological evaluation of the natural product komaroviquinone and related compounds aiming at a potential therapeutic lead compound for high-risk multiple myeloma. Bioorg. Med. Chem. Lett..

[190] Weremczuk-Jeżyna I., Kuźma Ł., Grzegorczyk-Karolak I. (2021). The effect of different light treatments on morphogenesis, phenolic compound accumulation and antioxidant potential of *Dracocephalum forrestii* transformed shoots cultured in vitro. J. Photochem. Photobiol. B.

[191] Weremczuk-Jeżyna I., Kochan E., Szymczyk P., Lisiecki P., Kuźma Ł., Grzegorczyk-Karolak I. (2019). The antioxidant and antimicrobial properties of phenol-rich extracts of *Dracocephalum forrestii* W.W. Smith shoot cultures grown in the nutrient sprinkle bioreactor. Phytochem. Lett..

[192] Weremczuk-Jeżyna I., Lisiecki P., Gonciarz W., Kuźma Ł., Szemraj M., Chmiela M., Grzegorczyk-Karolak I. (2020). Transformed Shoots of *Dracocephalum forrestii* W.W. Smith from Different Bioreactor Systems as a Rich Source of Natural Phenolic Compounds. Molecules.

[193] Weremczuk-Jeżyna I., Lebelt L., Piotrowska D.G., Gonciarz W., Chmiela M., Grzegorczyk-Karolak I. (2023). The optimization growth of *Dracocephalum forrestii* in RITA^®^ bioreactor, and preliminary screening of the biological activity of the polyphenol-rich extract. Acta Sci. Pol. Hortorum Cultus.

[194] Zhang R.-Q., Ma X.-L., Chen Y.-P., Xiang C.-L. (2024). Complete chloroplast genome sequences of *Dracocephalum argunense* and *D. integrifolium* (Lamiaceae: Nepetinae). J. Asia-Pac. Biodivers..

[195] Kim S.-H., Shin T.-Y. (2006). Effect of *Dracocephalum argunense* on mast-cell-mediated hypersensitivity. Int. Arch. Allergy Immunol..

[196] Kim S.-H., Kim S.-H., Kim S.-H., Moon J.-Y., Park W.-H., Kim C.-H., Shin T.-Y. (2006). Action of *Dracocephalum argunense* on mast cell-mediated allergy model. Biol. Pharm. Bull..

[197] Ma S., Jia J., Wu L., Tian K., Wang L., Li H., Lv J., Gao D., Yang Z., Guo X. (2025). Total flavonoids of *Dracocephalum moldavica* L. alleviate cognitive impairment via TNF-α/NF-κB p65 signaling pathway in vascular dementia rats. Front. Pharmacol..

[198] Shanaida M., Lipka K., Kucher T., Kryskiw L., Pryshlyak A., Koval M. (2025). Phytochemical profiling and antioxidant activity of *Dracocephalum officinale* (blue-flowered form) cultivated in Ukraine. Biomed. Pharmacol. J..

[199] Yazdiniapour Z., Rashidi N., Safaeian L., Karimian P. (2026). *Dracocephalum subcapitatum* alleviates hyperglycemia, dyslipidemia, oxidative stress, and organ injury in STZ-induced diabetic rats: A dose-response study. Res. J. Pharmacogn..

[200] Chen Y.-P., Chen Y.-S., Xiang C.-L. (2021). *Dracocephalum microphyton* (Lamiaceae: Nepetoideae), a new species from south-west China. Kew Bull..

[201] Gafitskaya I.V., Bezdelev A.B., Nakonechnaya O.V. (2022). *Dracocephalum charkeviczii Prob.* in vitro. Bot. Pac..

[202] Tan H., Fu J., Chen C., Xiao L.-Q., Zhang J.-Z., Zhu T.-T., Ni R., Du N.-H., Ta H., Hao Y. (2025). Unraveling the hydroxylation and methylation mechanism in polymethoxylated flavones biosynthesis in *Dracocephalum moldavica*. Plant Physiol. Biochem..

[203] Frąc M., Oszust K., Kocira A., Kocira S. (2015). Molecular identification of fungi isolated from *Dracocephalum moldavica* L. seeds. Agric. Agric. Sci. Procedia.

[204] Ferreira E.d.O., Fernandes M.Y.S.D., de Lima N.M.R., Neves K.R.T., do Carmo M.R.S., Lima F.A.V., Fonteles A.A., Menezes A.P.F., de Andrade G.M. (2016). Neuroinflammatory response to experimental stroke is inhibited by eriodictyol. Behav. Brain Res..

[205] Kiani H.S., Sabokdast M., Dedicova B. (2025). Influence of Biotic and Abiotic Elicitors on Rosmarinic Acid Accumulation in Hairy Root Cultures of *Dracocephalum kotschyi* Boiss. Plants.

[206] Liu C., Li Y., Zhang Y., Li S., Dang J. (2026). Functional constituents of *Dracocephalum heterophyllum* recognized by HPLC-tyrosinase: DFT calculation, molecular docking, and in vivo activity studies. J. Mol. Struct..

[207] Ashrafian S., Moridi Farimani M., Sonboli A., Ashrafian H., Kabiri M., Rezadoost H. (2022). Simultaneous characterization of nine isolated flavonoids in Iranian *Dracocephalum* species and in silico study of their inhibitory properties against MTH1 enzyme. S. Afr. J. Bot..

[208] Taghizadeh M., Khosravi Khouzani Z., Yousefian Z. (2026). Chitosan nanoparticles as elicitors of phenolic compounds in cell cultures of *Dracocephalum polychaetum* Boiss.: A dose-dependent in vitro study. Plant Nano Biol..

[209] Behzadi M., Jarollahi S., Ahsani Irvani M., Ghanbari D. (2022). Green synthesis and antibacterial activity of silver nanoparticles using *Dracocephalum moldavica* leaves extract. J. Nanostruct..

[210] Taghizadeh M., Nasibi F., Manouchehri Kalantari K., Benakashani F. (2020). Callogenesis optimization and cell suspension culture establishment of *Dracocephalum polychaetum* Bornm. and *Dracocephalum kotschyi* Boiss.: An in vitro approach for secondary metabolite production. S. Afr. J. Bot..

[211] Horn T., Völker J., Rühle M., Häser A., Jürges G., Nick P. (2014). Genetic authentication by RFLP versus ARMS? The case of Moldavian dragonhead (*Dracocephalum moldavica* L.). Eur. Food Res. Technol..

[212] Rahmati E., Sharifian F., Fattahi M. (2020). Process optimization of spray-dried Moldavian balm (*Dracocephalum moldavica* L.) extract powder. Food Sci. Nutr..

[213] Sheychenko O.P., Sheychenko V.I., Goryainov S.V., Zvezdina E.V., Kurmanova E.N., Ferubko E.V., Uyutova E.V., Potanina O.G., Fadi K. (2022). Chemical composition and biological activity of Rosmatin, a dry extract from *Dracocephalum moldavica* L.. Russ. J. Bioorg. Chem..

[214] Fathi S., Movlodzadeh R., Heidari S., Najafian S. (2025). Application of machine learning models to predict secondary metabolites for the first time in the valuable medicinal plant (*Dracocephalum moldavica* L.). Res. Sq..

[215] Zhao Q., Zhang X., Wang W., Yu H., Wang Z. (2026). Multi-omics analyses of *Dracocephalum moldavica* L. reveal two flavonoid glycosyltransferases in tilianin biosynthesis. BMC Genom..

[216] Omidbaigi R., Borna F., Borna T., Inotai K. (2009). Sowing dates affecting on the essential oil content of dragonhead (*Dracocephalum moldavica* L.) and its constituents. J. Essent. Oil Bear. Plants.

[217] Rezaie Keikhaie K., Jahantigh H.R., Bagheri R., Rezaie Kehkhaie A. (2018). The effects of the ethanol extract of *Dracocephalum moldavica* (Badrashbu) against strains of antibiotic-resistant *Escherichia coli* and *Klebsiella pneumonia*. Int. J. Infect..

[218] Yu H., Chen Z., Chen H., Wang Z. (2024). *Dracocephalum moldavica* L. extract ameliorates hypertension through modulating the interaction between miRNAs and gut microbiota in 2K1C rats. Nat. Prod. Commun..

[219] Sun M., Guo M., He Z., Luo Y., He X., Huang C., Yuan Y., Zhao Y., Song X., Wang X. (2024). Enhanced Anti-Inflammatory Activity of Tilianin Based on the Novel Amorphous Nanocrystals. Pharmaceuticals.

[220] Chitiala R.-D., Lungu I.I., Marin G.-A., Mitran A.-M., Caba I.-C., Lungu A., Robu S., Mircea C., Stefanache A., Hancianu M. (2025). Comparative Analysis of *Lespedeza* Species: Traditional Uses and Biological Activity of the Fabaceae Family. Molecules.

[221] Ghavam M., Manconi M., Manca M.L., Bacchetta G. (2021). Extraction of essential oil from *Dracocephalum kotschyi* Boiss. (Lamiaceae), identification of two active compounds and evaluation of the antimicrobial properties. J. Ethnopharmacol..

[222] Baumann J., Bönzli E., Wandrey F., Grothe T. (2025). Moldavian dragonhead extract: A natural collagen-booster to target skin aging. OBM Geriatr..

[223] Guo M., Wei S., Cheng B., Li X. (2026). *Dracocephalum tanguticum* Maxim polysaccharide ameliorate autoimmune hepatitis via regulating TNF and IL-17 signaling pathways. Food Sci. Nutr..

[224] Ibrahim F.M., Ibrahim M.M., Abbassinya H., Rostami Cheri F., Nazarpoy Shirehjini S., Farahbod F., Khademi N. (2022). The Effect of *Dracocephalum* Extract on Sleep Quality in Post-Menopausal Women: A Randomized Placebo-Controlled Trial. Iran. J. Psychiatry.

[225] Poursalavati A., Rashidi-Monfared S., Ebrahimi A. (2021). Toward understanding of the methoxylated flavonoid biosynthesis pathway in *Dracocephalum kotschyi* Boiss. Sci. Rep..

[226] Maimaiti A., Tao Y., Wang M., Weiwei M., Wenhui S., Aikemu A., Maimaitiyiming D. (2021). Improvement of Total Flavonoids from *Dracocephalum moldavica* L. in Rats with Chronic Mountain Sickness through 1H-NMR Metabonomics. Evid.-Based Complement. Altern. Med..

[227] Zhao Q., Fan Z., Yu H., Wang Z. (2025). The high-quality chromosome-level genome assembly of *Dracocephalum rupestre* Hance. Sci. Data.

[228] Xiong H.-H., Lin S.-Y., Chen L.-L., Ouyang K.-H., Wang W.-J. (2023). The Interaction between Flavonoids and Intestinal Microbes: A Review. Foods.

[229] Cichon N., Szelenberger R., Stela M., Podogrocki M., Gorniak L., Bijak M. (2025). Flavanones as Modulators of Gut Microbiota and Cognitive Function. Molecules.

[230] Li K., Wu J., Xu S., Li X., Zhang Y., Gao X.-J. (2023). Rosmarinic acid alleviates intestinal inflammatory damage and inhibits endoplasmic reticulum stress and smooth muscle contraction abnormalities in intestinal tissues by regulating gut microbiota. Microbiol. Spectr..

[231] Zheng R.-F., Du Y.-W., Zeng C., Wang H.-F., Xing J.-G., Xu M. (2020). Total flavones of *Dracocephalum moldavica* L. protect astrocytes against H_2_O_2_-induced apoptosis through a mitochondria-dependent pathway. BMC Complement. Med. Ther..

[232] Kim K.-M., Kim S.-Y., Mony T.J., Bae H.J., Han S.-D., Lee E.-S., Choi S.-H., Hong S.H., Lee S.-D., Park S.J. (2021). *Dracocephalum moldavica* ethanol extract suppresses LPS-induced inflammatory responses through inhibition of the JNK/ERK/NF-κB signaling pathway and IL-6 production in RAW 264.7 macrophages and in endotoxic-treated mice. Nutrients.

[233] Zverev Y.F., Rykunova A.Y. (2022). Modern Nanocarriers as a Factor in Increasing the Bioavailability and Pharmacological Activity of Flavonoids. Appl. Biochem. Microbiol..

[234] Khanizadeh P., Mumivand H., Morshedloo M.R., Maggi F. (2024). Application of Fe_2_O_3_ nanoparticles improves the growth, antioxidant power, flavonoid content, and essential oil yield and composition of *Dracocephalum kotschyi* Boiss. Front. Plant Sci..

[235] Zvezdina E.V., Sheychenko O.P., Burova A.E. (2020). Development of a procedure for standardization of the Rozmatin substance from Moldavian dragonhead (*Dracocephalum moldavice*) herb. Farmatsiya.

[236] Sadraei H., Rasouli-Amirabadi A.H., Yegdaneh A., Tavakoli N. (2022). Bioassay standardization of drug dosage form prepared from hydroalcoholic extract of *Dracocephalum kotschyi*. J. Herbmed Pharmacol..

